# FEGS: a novel feature extraction model for protein sequences and its applications

**DOI:** 10.1186/s12859-021-04223-3

**Published:** 2021-06-03

**Authors:** Zengchao Mu, Ting Yu, Xiaoping Liu, Hongyu Zheng, Leyi Wei, Juntao Liu

**Affiliations:** 1 School of Mathematics and Statistics, Shandong University, Weihai, 264209 China; 2 Research Center for Mathematics and Interdisciplinary Sciences, Shandong University, Qingdao, 266237 China; 3 Hangzhou Institute for Advanced Study, University of Chinese Academy of Sciences, Beijing, China; 4 Department of Radiation Oncology, Qilu Hospital, Cheeloo College of Medicine, Shandong University, Jinan, 250012 China; 5 School of Software, Shandong University, Jinan, China

**Keywords:** Feature extraction, Graphical representation, Physicochemical properties of amino acids, Statistical features, Protein similarity analysis

## Abstract

**Background:**

Feature extraction of protein sequences is widely used in various research areas related to protein analysis, such as protein similarity analysis and prediction of protein functions or interactions.

**Results:**

In this study, we introduce FEGS (Feature Extraction based on Graphical and Statistical features), a novel feature extraction model of protein sequences, by developing a new technique for graphical representation of protein sequences based on the physicochemical properties of amino acids and effectively employing the statistical features of protein sequences. By fusing the graphical and statistical features, FEGS transforms a protein sequence into a 578-dimensional numerical vector. When FEGS is applied to phylogenetic analysis on five protein sequence data sets, its performance is notably better than all of the other compared methods.

**Conclusion:**

The FEGS method is carefully designed, which is practically powerful for extracting features of protein sequences. The current version of FEGS is developed to be user-friendly and is expected to play a crucial role in the related studies of protein sequence analyses.

**Supplementary Information:**

The online version contains supplementary material available at 10.1186/s12859-021-04223-3.

## Background

The similarity analysis of protein sequences is one of the major topics in bioinformatics. It has many applications in the study of protein evolution and functions, as well as gene annotation, gene function prediction, identification and construction of gene families, and gene discovery [1].

With the number of available protein sequences developing rapidly, plenty of approaches have been proposed for protein sequence similarity analysis. These approaches can be generally divided into two categories: alignment-based methods and alignment-free methods. Blast [2] and Clustal [3] are two most widely used algorithms for sequence alignment. Although alignment-based methods achieve satisfactory results in sequence comparison, they often involve in high computational complexity. In addition, alignment-based methods have been shown to be inaccurate in scenarios of low sequence identity [4]. In order to overcome the limitations of alignment-based methods, many alignment-free ones are proposed for sequence comparison. Generally, the alignment-free methods first transform a protein sequence into a numerical vector, and then calculate the distance between the numerical vectors as a measure of sequence similarity. This transformation from sequence to numerical vector is called feature extraction of protein sequences, which is a key step for the alignment-free methods. However, extracting effective protein features based only on the primary sequences is a highly challenging task. To date, various protein feature extraction approaches have been developed for encoding protein sequences and extracting hidden information, among which the graphical representation is one of the most efficient and widely used strategies. The advantage of the graphical representation is that it allows direct visualization of protein sequences. Moreover, the generated graphical curve can be associated with a matrix, such as matrices E, M/M, and L/L [5–8]. Then, the invariants derived from the matrix can be used as the numerical descriptors to analyze the sequence similarity [9–14].

Biological molecule graphical representation was first introduced and applied to representing DNA sequences by Hamori and Ruskin in 1983, in which a DNA sequence was transformed into a three dimensional graphical curve [15]. Since then, many different models of graphical representation of DNA and protein sequences have been developed [16–28]. In the graphical representations of DNA sequences, the 4 nucleotides were first represented by 4 pre-given vectors, and then an iterated function system (IFS) was used to transform a DNA sequence into a space curve based on these vectors. In contrast to DNA sequences, which contain only 4 nucleotides, protein sequences are made up of 20 amino acids. The substitution from 4 bases to 20 amino acids brings computational difficulties to the graphical representations of protein sequences. To address the difficulty of processing 20 amino acid letters for protein sequences, Li [5], Yu [29], Manikandakumar [30], He [31], Yao [32] and Basu [33] used reduced amino acid alphabet to build graphical representations of protein sequences, in which the 20 amino acids were classified into 4, 5, 6, 8 or 12 groups according to their physicochemical properties, respectively. Then, each protein sequence was correspondingly transformed into a 4-, 5-, 6-, 8- or 12-letter sequence, based on which the graphical representation of protein sequences was performed. However, using a reduced amino acid alphabet to represent protein sequences easily results in loss of sequence information, since different amino acids belonging to the same group are considered identical. The physicochemical properties of amino acids are important for protein structures, functions and protein–protein interactions and have strong effects on the pattern of protein evolution. In [34], Randić mentioned that ordering amino acids based on their physicochemical properties may offer better insights in comparative studies of proteins than representations of proteins based on alphabetical ordering of amino acids. Therefore, physicochemical properties of amino acids have been widely used in protein sequence studies. According to the physicochemical properties of amino acids, He [11, 35], Wu [24], Yu [36, 37], Gupta [38], Yau [39], and Yao [40] proposed different graphical representation methods based on 20 amino acid characters. Each of the above methods used only a few physicochemical properties of amino acids, and therefore, a protein sequence only corresponded to one or a few graphical curves, which reduces the ability of the subsequent numerical descriptors to describe the protein sequence.

**Fig. 1:**
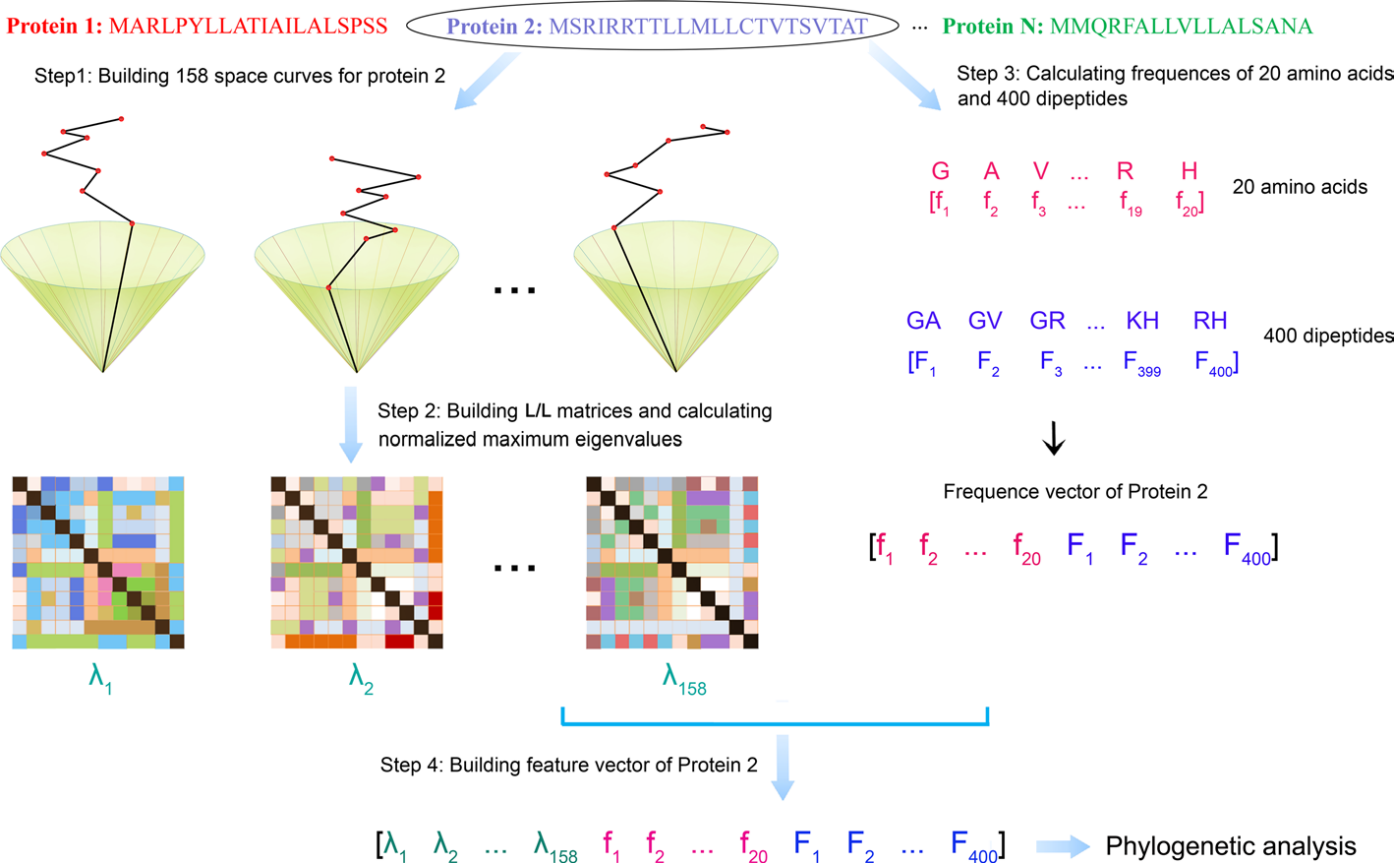
Flowchart of the method FEGS

In this paper, we introduce FEGS, a novel feature extraction method of protein sequences, by developing a new technique for the graphical representation of protein sequences based on the full use of physicochemical properties of amino acids and statistical information in the protein sequences. By integrating the graphical and statistical features of protein sequences, we finally obtained a 578-dimensional vector as the feature vector for each protein sequence (see Fig. 1 and Methods for details). To validate the effectiveness of FEGS, we applied it for phylogenetic analysis on five protein sequence data sets, and the results show that FEGS produces the most accurate phylogeny in all data sets among all the compared methods.

## Results

To fully demonstrate the validity of our method, we applied FEGS for phylogenetic analysis on five commonly used protein sequence data sets. For comparison, we also used five other feature extraction methods, *k*-mer natural vector [41], PseAAC [42], averaged property factors [43], natural vector [44] and protein map [45] to perform phylogenetic analysis on the same data sets.

### Phylogenetic analysis of 50 beta-globin protein sequences

**Fig. 2:**
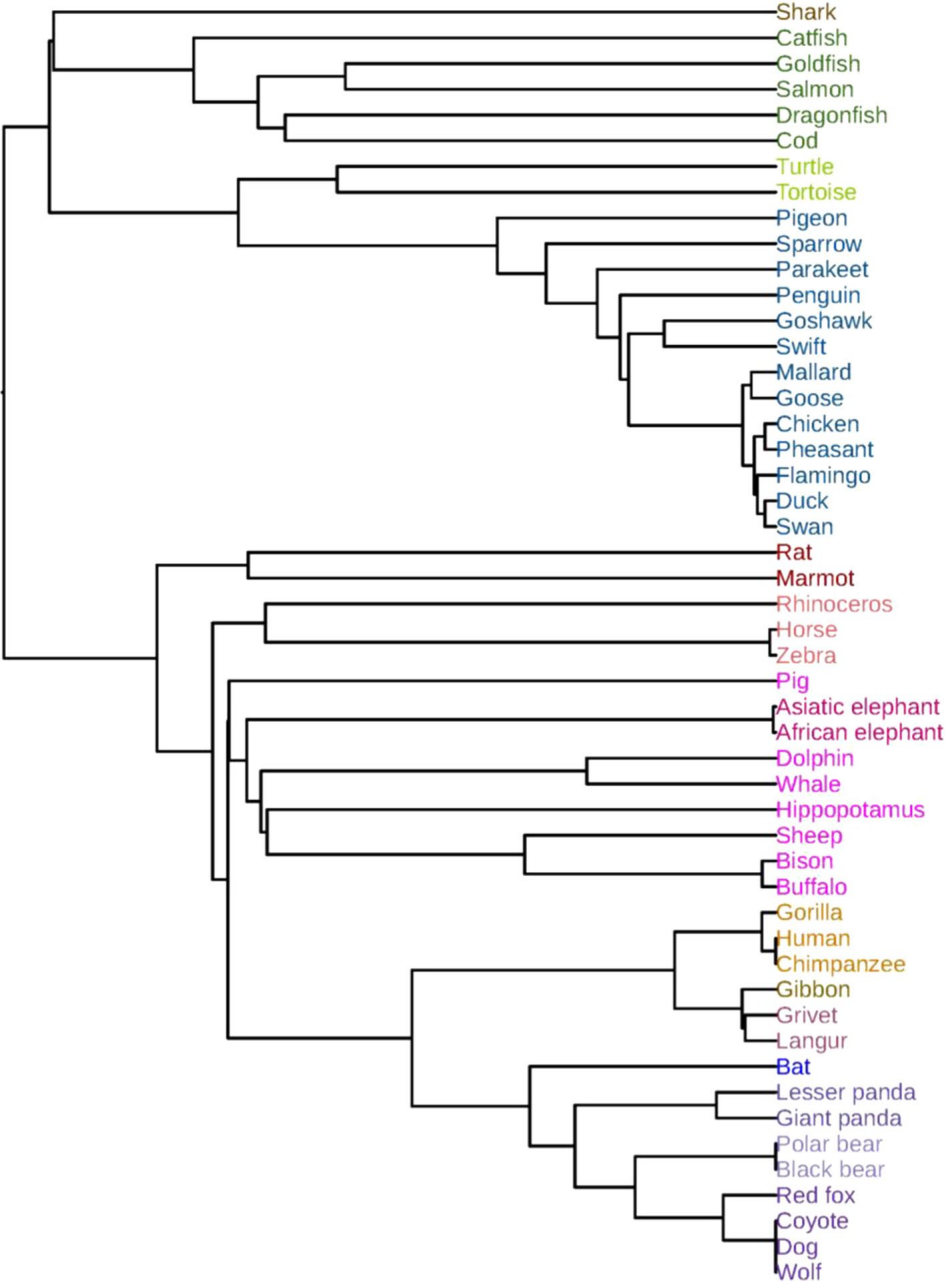
Phylogenetic tree of the 50 beta-globins constructed by FEGS using the single linkage method

This data set contains 50 beta-globin protein sequences from 50 species studied in [39, 46–48], and the accession numbers are shown in Additional file 1: Notes 1.2. After applying FEGS to the 50 protein sequences, we obtained a 50 × 578 feature matrix. Then, the PCA technique was applied to the matrix for dimension reduction, and the first 28 principal components were extracted as the feature vectors of the 50 protein sequences. The cosine distance was used to calculate the distance matrix of the 50 beta-globin protein sequences, and the phylogenetic tree was constructed by using the single linkage method and shown in Fig. 2.

As shown in Fig. 2, the 50 beta-globin proteins were clearly grouped into two main clusters: mammals and non-mammals. In the mammalian cluster, the beta-globin proteins belonging to Carnivora (lesser panda, giant panda, black bear, polar bear, coyote, wolf, red fox, dog), Primate (human, gorilla, chimpanzee, grivet, langur and gibbon), Rodentia (rat, marmot), Proboscidea (Asiatic elephant, African elephant), and Perissodactyla (horse, rhinoceros, zebra) are accurately separated and grouped into respective taxonomic classes. Except for pig, all species belonging to the Artiodactyla (hippopotamus, whale, dolphin, sheep, bison, buffalo) are also clustered into one branch. Furthermore, the beta-globin proteins belonging to Canidae (coyote, wolf, red fox, dog) in Carnivora and Ruminantia (sheep, bison, buffalo) in Artiodactyla are also accurately grouped together, respectively. Hominidae (human, gorilla, chimpanzee), Cercopithecidae (grivet, langur) and Hylobatidae (gibbon) in Primate are clearly divided into three separate sub-branches. In the nonmammalian cluster, the beta-globin proteins belonging to aves, fish and reptile were also perfectly separated and grouped into respective taxonomic classes. In the branch of fishes, the Chondrichthyes (shark) are correctly separated from the Actinopterygii (Dragonfish, cod, goldfish, salmon and catfish), which is also consistent with the known evolutionary relationships.

The phylogenetic trees constructed by the other five feature extraction methods (*k*-mer natural vector, PseAAC, averaged property factors, natural vector and protein map) using the single linkage method are respectively shown in Additional file 1: Figs. S1–S5. In Additional file 1: Fig. S1, the beta-globin proteins of Artiodactyla and those of Rodentia, Perissodactyla and Proboscidea are mixed together and not separated. In Additional file 1: Fig. S2, the beta-globin proteins of Artiodactyla are also not clustered together, and the Rat and Marmot belonging to the Rodentia are clustered into non-mammalian branches. The proteins of Perissodactyla are also not clustered together. In Additional file 1: Fig. S3, rat and marmot are erroneously clustered into the branch of aves. Neither the Artiodactyla nor the Perissodactyla are clustered into separate branches. In Additional file 1: Fig. S4, asiatic elephant, african elephant, rat, pig and whale are erroneously clustered into the branch of fishes. Salmon is erroneously clustered into the mammalian branch. The Carnivora, Primate and Artiodactyla are not clustered into separate branches. In Additional file 1: Fig. S5, turtle and tortoise are erroneously clustered into the branch of fishes. Rat, rhinoceros, horse and zebra are also clustered incorrectly.

### Phylogenetic analysis of 27 AFPs

**Fig. 3:**
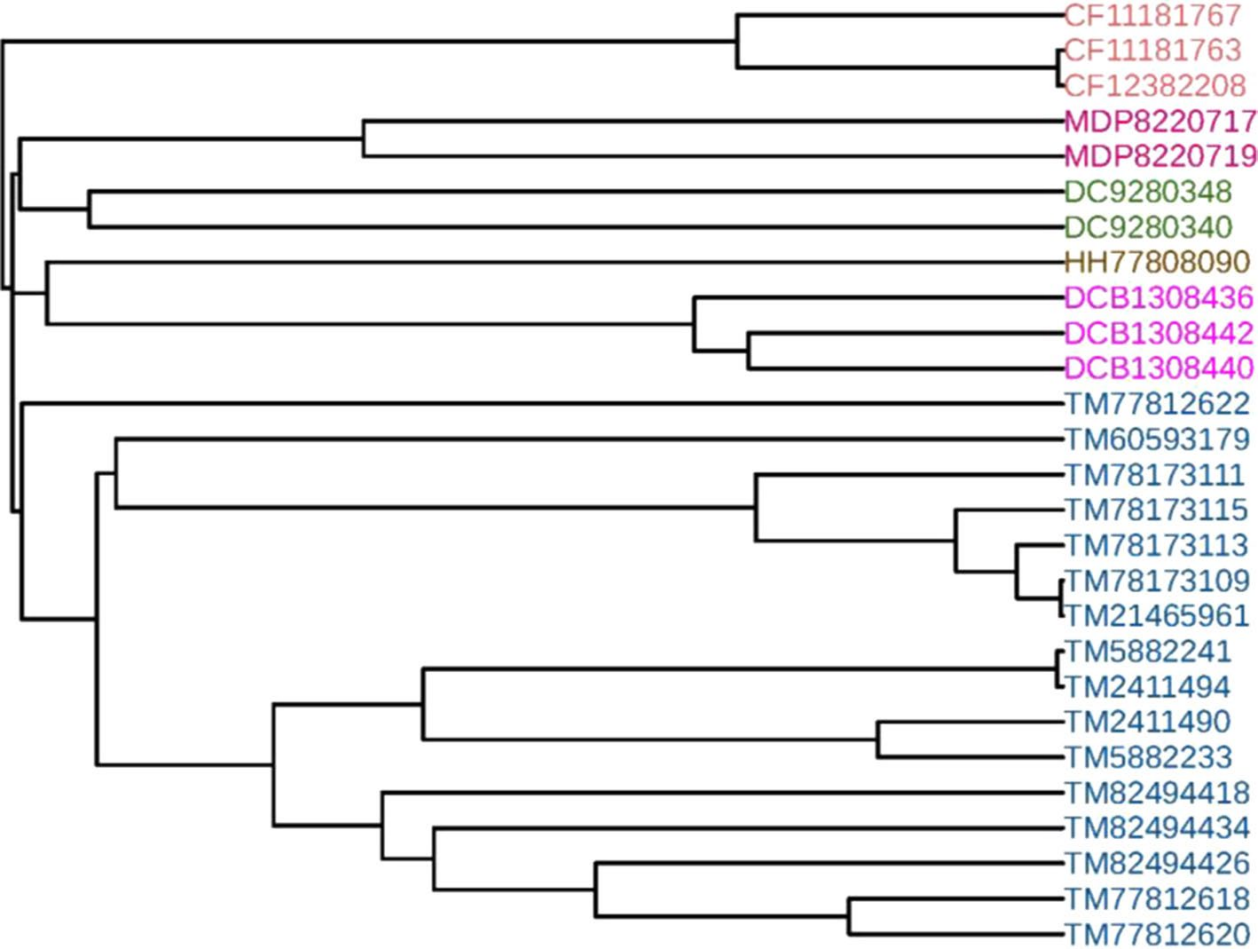
Phylogenetic tree of the 27 AFPs constructed by FEGS using the single linkage method

On this data set, 27 antifreeze protein sequences (AFPs) studied in [46, 48–50] were collected to verify the effectiveness of our method. The 27 AFPs were selected from *Choristoneura fumiferana* (CF), *Tenebrio molitor* (TM), *Hypogastrura harveyi* (HH), *Dorcus curvidens binodulosus* (DCB), *Microdera dzhungarica punctipennis* (MDP) and *Dendroides canadensis* (DC), and the taxonomic information and accession numbers of the 27 proteins are provided in Additional file 1: Table S1. The phylogenetic tree of the 27 AFPs was constructed by FEGS using the single linkage method and shown in Fig. 3, which clearly shows that the AFPs belonging to the same species were accurately clustered together and form separate branches.

The phylogenetic trees constructed by the other five feature extraction methods (*k*-mer natural vector, PseAAC, averaged property factors, natural vector and protein map) using the single linkage method are shown in Additional file 1: Fig. S6-S10, respectively. From Additional file 1: Fig. S6-S10, it shows that all the five methods erroneously clustered the antifreeze proteins of TM, MDP, DCB and DC.

### Phylogenetic analysis of 40 coronavirus spike protein sequences

**Fig. 4:**
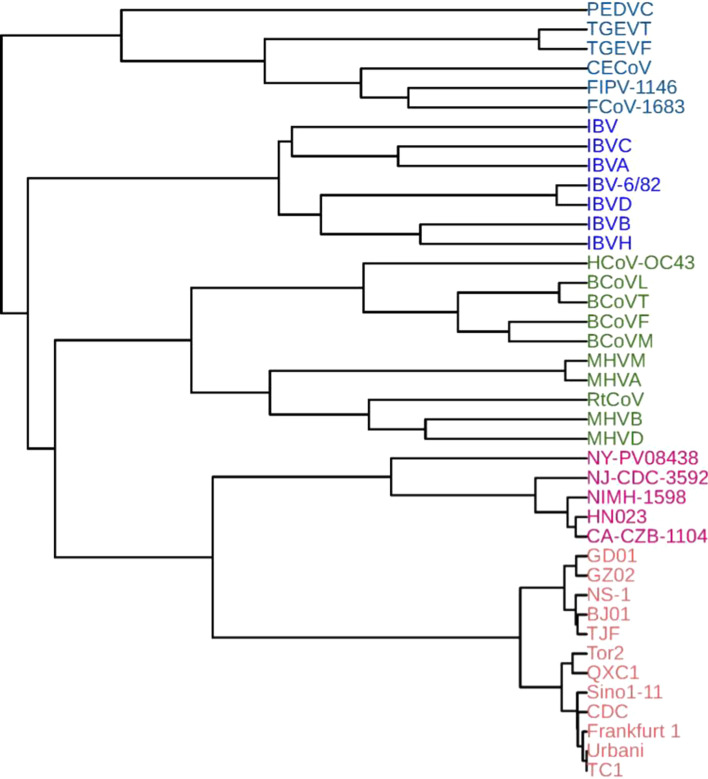
Phylogenetic tree of the 40 coronavirus spike proteins constructed by FEGS using the complete linkage method

FEGS was also applied for performing phylogenetic analysis on a data set consisting of 40 coronavirus spike protein sequences. This data set is obtained by adding 5 spike protein sequences of 2019 novel coronavirus (2019-nCoV) to the data set containing 35 coronavirus spike protein sequences studied in [51, 52]. The taxonomic information and accession numbers of the 40 protein sequences are shown in Additional file 1: Table S2. According to the taxonomic groups, sequences 1–6 belong to group alpha, sequences 7–13 are members of group gamma, and the remaining belongs to group beta. The corresponding phylogenetic tree constructed by FEGS using the complete linkage method is shown in Fig. 4, which accurately clustered the coronaviruses into three separate branches. Moreover, in the branch of the group alpha, the spike proteins of Alphacoronavirus 1 ((FIPV-1146, FCoV-1683), CECoV, (TGEVF, TGEVT), PEDVC) are correctly clustered together, and in the branch of the group beta, the spike proteins of Betacoronavirus 1 ((BCoVF, BCoVM, BCoVL, BCoVT), HCoV-OC43), Murine coronavirus (MHVM, MHVB, MHVA, MHVD, RtCoV), SARS-CoV (Tor2, BJ01, NS-1, GD01, Frankfurt 1, Urbani, TC1, CDC, GZ02, QXC1, Sino1-11, TJF) and SARS-CoV-2 (NIMH-1598, HN023, NY-PV08438, NJ-CDC-3592, CA-CZB-1104) are all accurately clustered into separate branches. In addition, the phylogenetic tree in Fig. 4 clearly shows that the 2019-nCoVs are more closely related to SARS-CoVs than to Betacoronavirus 1 and Murine coronaviruses, which is consistent with the result reported in [53].

The phylogenetic trees constructed by the other five feature extraction methods (*k*-mer natural vector, PseAAC, averaged property factors, natural vector and protein map) using the complete linkage method are shown in Additional file 1: Figs. S11–S15, respectively. In Additional file 1: Fig. S11 and S12, the spike proteins of Betacoronavirus are not clustered together and form a separate branch. In Additional file 1: Fig. S13 and S14, PEDVC was not clustered into the branch of Alphacoronavirus 1. NY-PV08438 are erroneously clustered in Additional file 1: Fig. S14 and S15.

### Phylogenetic analysis of 25 transferrin sequences

**Fig. 5:**
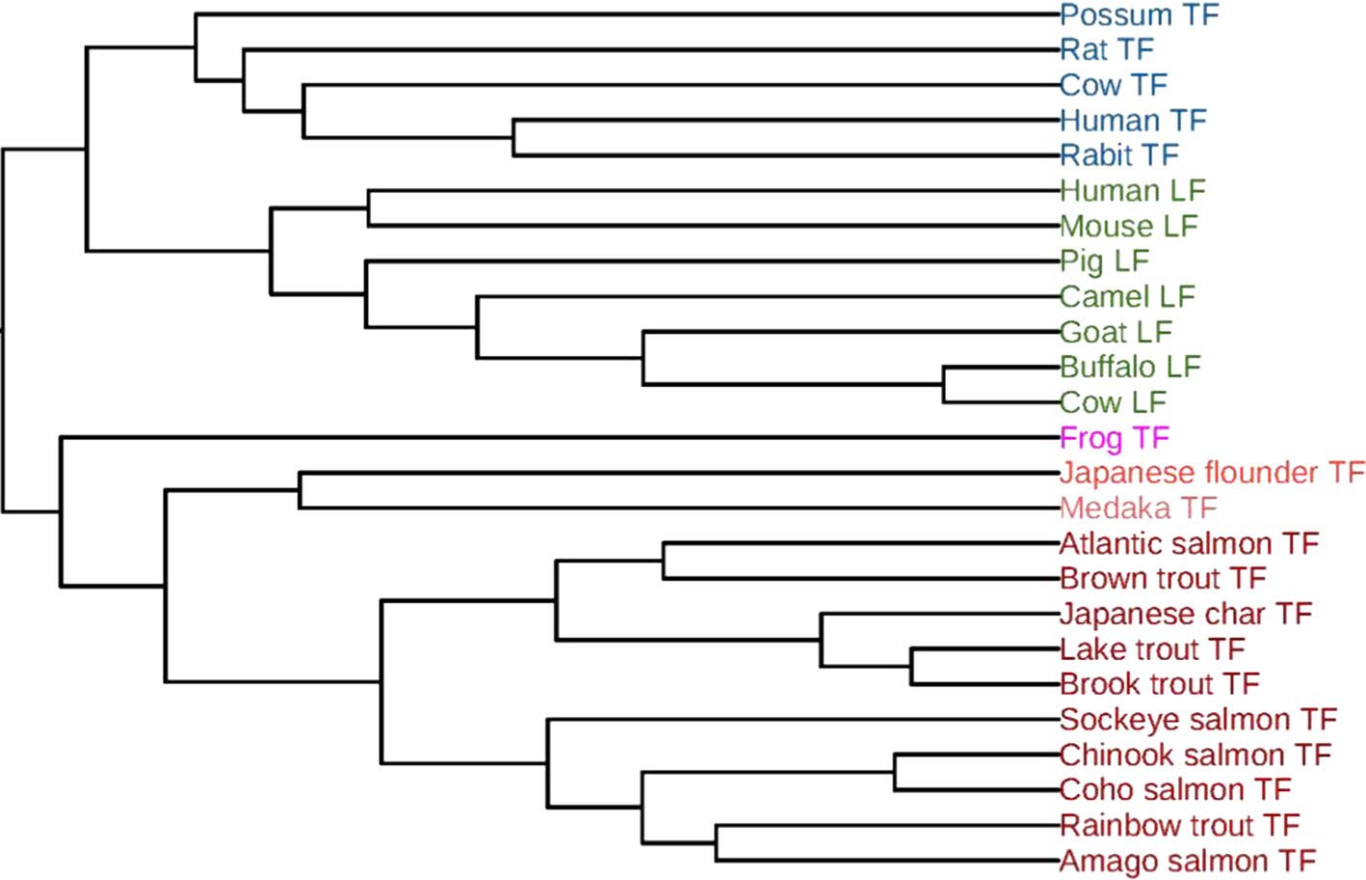
Phylogenetic tree of the 25 TFs constructed by FEGS using the complete linkage method

The phylogenetic analysis by using FEGS was also performed on the data set containing 25 transferrin sequences (TFs) from 25 vertebrates, which was studied in [46, 54]. The taxonomic information and accession numbers of the 25 proteins are shown in Additional file 1: Table S3. The phylogenetic tree of the 25 TFs constructed by our method using the complete linkage method is shown in Fig. 5. From the Fig. 5, it is clear that all TFs are accurately grouped into three branches: fish, amphibian and mammal. In the branch of mammals, transferrin (TF) proteins and lactoferrin (LF) proteins are correctly separated and clustered into different branches. In the branch of LFs, the LFs of the Artiodactyla (Buffalo LF, Cow LF, Goat LF, Camel LF, Pig LF) are clustered together and form a separate branch. In the group of fish, all the TFs from Salmonidae are clustered together and form a separate branch. In addition, the TFs belonging to Salmo (Atlantic salmon TF, Brown trout TF), Salvelinus (Lake trout TF, Brook trout TF, Japanese char TF) and Oncorhynchus (Chinook salmon TF, Coho salmon TF, Sockeye salmon TF, Rainbow trout TF, Amago salmon TF) are also correctly clustered together and form separate branches, respectively. All these results are completely consistent with the known evolutionary relationships.

The phylogenetic trees constructed by the other five feature extraction methods (*k*-mer natural vector, PseAAC, averaged property factors, natural vector and protein map) using the complete linkage method are shown in Additional file 1: Figs. S16–S20, respectively. In Additional file 1: Fig. S16 and S18, the LFs of the Artiodactyla are not clustered together. In Additional file 1: Fig. S17, the TFs of mammal and fish are erroneously clustered together. In S19 and S20, the TFs and LFs are mixed together without being separated, and the TFs of rat, human and rabit are erroneously clustered into the branch of fish.

### Phylogenetic analysis of Human rhinovirus

**Fig. 6:**
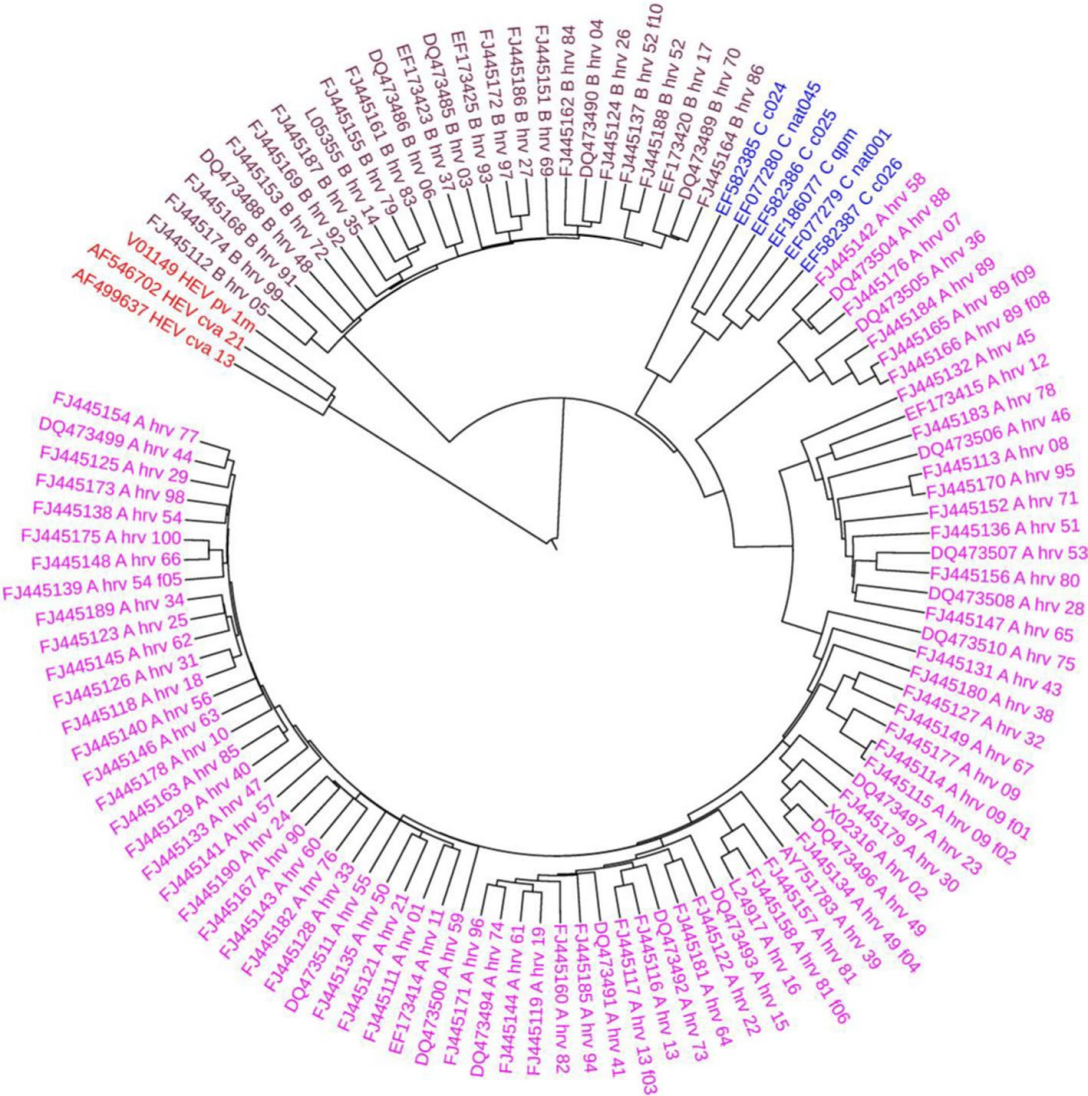
Phylogenetic tree of the 111 HRVs and 3 HEV-Cs constructed by FEGS using the single linkage method

Finally, FEGS was applied for phylogenetic analysis on a data set consisting of 111 HRV and 3 HEV-C proteins. Human rhinovirus (HRV) is one of the most important causes of respiratory infections and has been associated mostly with the common cold [41]. It belongs to genus Enterovirus and family Picornaviridae. The phylogenetic analysis of the whole genome of this data set show that the HRVs can be classified into three distinct groups, HRV-A, HRV-B, and HRV-C, and HRV-A and HRV-C share a common ancestor, which is a sister group of HRV-B, and 3 HEV-C sequences formed an outgroup [55]. The phylogenetic tree constructed by FEGS using the single linkage method is shown in Fig. 6. As shown in Fig. 6, all 111 HRVs are clustered into three groups: HRV-A, HRV-B, and HRV-C, and 3 HEV-Cs form an outgroup, which are in accord with clinical heterogeneity of HRV infections in humans and the result reported in [55].

The phylogenetic trees constructed by the other five feature extraction methods (*k*-mer natural vector, PseAAC, averaged property factors, natural vector and protein map) using the single linkage method are shown in Additional file 1: Fig. S21-S25, respectively. The results in Additional file 1: Fig. S21 and S25 are similar with those of FEGS. In Additional file 1: Fig. S22, S23, S24, HRV-A and HRV-Cn are not clustered together.

### Comparison of clustering accuracy

According to the phylogenetic trees constructed by the feature extraction methods, we clustered protein sequences into *k* clusters for each method, where *k* is equal to the number of clusters in each data set based on taxonomic classification (see Additional file 1: Notes 1.3 for the details). Then the Adjusted Rand Index (ARI) [56] between the clustering by each feature extraction method and the clustering based on taxonomic classification is used as a measure for evaluating the classification accuracy of the feature extraction methods on all the five data sets in this paper. After comparison, results showed that FEGS consistently achieved the highest classification accuracy among all the compared methods on the five data sets (see Table 1 for details).

**Table 1:** ARI values of the six feature extraction methods on the five data sets

Data set	FEGS	*k*-mer natural vector	PseAAC	Averaged property factor	Natural vector	Protein map
1	0.948	0.3036	0.1339	0.1339	0.1339	0.1974
2	0.8871	0.4618	0.1646	0.4618	0.4396	0.0889
3	1	0.5043	0.3834	0.8975	0.5684	0.9153
4	0.9554	0.8798	0.937	0.6111	0.6549	0.3489
5	1	1	0.2287	0.3035	0.3405	0.8475

## Discussion

In this paper, we presented a novel feature extraction model, FEGS, for protein sequence. After applying it for phylogenetic analyses on five protein sequence data sets, FEGS consistently showed the best performances over all the compared methods, which clearly demonstrates its strong effectiveness. The superiority of FEGS may be attributed to the following.

First, FEGS utilizes a novel technique for graphical representation of protein sequences by extending 3D protein paths based on different newly designed right circular cones in 3D space. The generated 3D curves effectively capture the global features of a protein and provide key information for subsequent feature extractions. Second, FEGS attempts to build multiple circular cones in 3D space by taking advantage of the physicochemical properties of amino acids and the accumulative frequencies of amino acid pairs in the protein sequence. Third, FEGS further integrates amino acid composition and dipeptide composition which have been widely used in protein sequence analysis, and finally generates a 578-dimensional vector as the numerical feature for each protein sequence.

Computational complexity is also important for feature extraction methods. Methods with similar accuracy but lower computation complexity are more favorable than methods with similar accuracy but higher computational complexity. Therefore, we compared the running time of each method on the same platform with a 16 GB memory and a 8-core CPU, and we found that all the methods are very efficient and cost similar running times. For example, on the first data set, the running time of FEGS for processing 50 protein sequences was 1.7 s, and the running times of *k*-mer natural vector, protein map, PseAAC, natural vector, and averaged property factors were 4.71 s, 0.99 s, 0.98 s, 0.93 s, and 0.96 s, respectively.

Although we have seen some promising results of FEGS, further improvements can still be made for FEGS in the future. For example, the current of FEGS cannot make use of the structural information of protein sequences for feature extraction. In addition, the values of the physicochemical properties of amino acids are only qualitatively used by FEGS for arranging the 20 amino acids on right circular cones, which is expected to enhance the performance of FEGS if they can be used quantitatively. Therefore, we will develop future versions for effectively employing protein structure information and quantitatively applying physicochemical properties of amino acids for more accurate feature extractions. In addition, as a feature extraction method, FEGS has potential applications in the fields of many prediction problems, which may be our future research areas. The current version of FEGS was developed to be user-friendly and is expected to play a crucial role in different researches related to protein sequence analysis.

## Conclusions

We in this study developed a practically effective method FEGS for extracting features from protein sequences. It is the first circular cone based method by effectively integrating the physicochemical properties of amino acids and the statistical features of protein sequences into the method design. Results show that FEGS is currently the most accurate method for protein feature extractions, and demonstrate great potentials for the studies of protein sequences related to similarity analyses, protein function predictions, protein–protein interactions, and so on.

## Methods

### AAindex database

The AAindex is a database of numerical indices representing various physicochemical and biochemical properties of amino acids and amino acid pairs [57, 58]. The latest version is the 9.2 release, which currently contains 566 indices. An amino acid index is a set of 20 numerical values representing any of the different physicochemical properties of the 20 amino acids. Here, we selected 158 indices for the following applications after removing all the redundant indices that have duplicate values. The 158 selected indices are detailed in Additional file 1: Notes 1.1.

### Construction of 3D graphical curves for protein sequences

Different from the approaches for representing protein sequences by using reduced amino acid alphabets, which easily lose protein sequence information, in this study, we developed a novel graphical representation method for protein sequences directly based on the 20 amino acids. First, the 20 amino acids are mapped to 20 points in 3D space according to their physicochemical indices selected from the AAindex database. Then each graphical curve of a protein sequence can be constructed by extending a 3D protein path based on a right circular cone.

### 1) Arrangement of the 20 amino acids and the 400 amino acid pairs

To make effective use of the physicochemical properties of amino acids, we first sorted the 20 amino acids according to their physicochemical indices in ascending order. Then, the 20 amino acids are arranged in order on the circumference of the bottom of a right circular cone with a height of 1 by the following equation:

$$\phi ({\Omega_i}) = \left( {\cos \frac{2\pi i}{{20}},\sin \frac{2\pi i}{{20}},1} \right),\quad i = 1,2, \ldots ,20$$

where Ω_*i*_ represents each of the 20 amino acids. Then, all 400 amino acid pairs are mapped to the underside of the right circular cone by the following equation:

$$\varphi ({\Omega_i}{\Omega_j}) = \phi ({\Omega_i}) + \frac{1}{4}(\phi ({\Omega_j}) - \phi ({\Omega_i})),\quad i,j = 1,2, \ldots ,20$$

where Ω_*i*_Ω_*j*_ corresponds to each of the 400 amino acid pairs.

### 2) Building 3D graphical curves for protein sequences

Given a protein sequence *S* with *N* amino acids *S* = *s*_1_*s*_2…_*s*_*N*_, its 3D graphical curve is constructed by extending a 3D protein path based on the above right circular cone as follows. Starting from the origin *P*_0_ (0, 0, 0), extend it to the next point *P*_1_ (*x*_1_, *y*_1_, *z*_1_) in 3D space corresponding to the first amino acid *s*_1_ and then to the point *P*_2_ (*x*_2_, *y*_2_, *z*_2_) corresponding to the second amino acid *s*_2_. The 3D protein path is extended until the path extension is completed at the last amino acid *s*_*N*_, and the 3D protein path *P* is obtained, corresponding to the 3D graphical curve of the protein sequence *S*. For the point *P*_i_ (*x*_i_, *y*_i_, *z*_i_) corresponding to the *i*th amino acid *s*_i_, its coordinates *x*_i_, *y*_i_, and *z*_i_ are determined by the following equation:

$$\psi ({S_i}) = \psi ({S_{i{ - }1}}) + \phi ({S_i}) + \sum\limits_{{\Omega_1},{\Omega_2} \in \{ A,C,D, \ldots ,Y\} } {{f_{{\Omega_1}{\Omega_2}}} \cdot \varphi ({\Omega_1}{\Omega_2})}$$

where $\psi \left({S}_{0}\right)=\left(\mathrm{0,0},0\right)$ and ${f}_{{\Omega }_{1}{\Omega }_{2}}$ is the frequency of the amino acid pair ${\Omega }_{1}{\Omega }_{2}$ in the subsequence of the first *i* amino acids of the protein sequence. Each of the 158 selected physicochemical properties corresponds to a unique right circular cone, and therefore, we can finally obtain 158 different 3D graphical curves for each protein sequence corresponding to the 158 different physicochemical properties of amino acids (see Fig. 1).

### Numerical features of protein sequences

After completing the graphical representation of protein sequences, the next task is to effectively transform the constructed curves into numerical characteristics, which can then be used for protein sequence similarity analysis. First, an L/L matrix *M* is computed for each graphical curve, which is a nonnegative symmetric matrix whose off-diagonal entries *M*_*i,j*_ (*i* ≠ *j*) are defined as a quotient of the Euclidean distance between two points *P*_*i*_ and *P*_*j*_ of the graphical curve and the sum of geometrical lengths of edges between *P*_*i*_ and *P*_*j*_ along the graphical curve, and all diagonal elements are equal to zero. Then, the leading eigenvalue of the matrix *M* is computed as the representative of the matrix to effectively characterize the corresponding graphical curve. To eliminate the biases of the lengths of different protein sequences, each leading eigenvalue is normalized by dividing the length of the corresponding protein sequence. After processing all 158 graphical curves for a protein sequence *S*, a 158-dimensional feature vector is generated as the graphical features of the corresponding protein sequence *S*, which can be formulated as follows (see Fig. 1):

$${V_g} = [{\lambda_1},{\lambda_2}, \ldots ,{\lambda_{158}}]$$

In addition to the graphical features from graphical representation above, we also investigated two commonly used statistical features: amino acid composition (AAC) and dipeptide composition (DPC), which are widely used in protein sequence analyses [59–64]. AAC reflects the occurrences of standard amino acids in a given protein sequence normalized by the sequence length. It has a fixed length of 20 features, which can be formulated as follows:

$${V_a} = [{f_1},{f_2}, \ldots ,{f_{20}}],$$

where *f*_*i*_ is the normalized frequency of the *i*-th amino acid in the protein sequence (see Fig. 1). DPC refers to the occurrence frequencies of the 400 amino acid pairs for a given protein sequence, which encapsulates the information of the amino acid fraction as well as the local order of amino acids in protein sequences. It has a fixed length of 400 elements, which can be formulated as follows:

$${V_d} = [{F_1},{F_2}, \ldots ,{F_{400}}]$$

where *F*_*j*_ represents the frequency of the *j*-th amino acid pair in {AA, AC, AD, AE, …,YY} (see Fig. 1).

The graphical features *V*_*g*_ and the statistical features *V*_*a*_ and *V*_*d*_ are merged into a 578-dimensional vector, which is taken as the final numerical features of the protein sequence *S* (see Fig. 1). Given a data set consisting of *N* protein sequences, we can obtain an *N* × 578 feature matrix, each row of which corresponds to a feature vector of a protein sequence. Since the dimension of the feature vectors is very high, there may be redundancies and noises in them. We use the Principal Component Analysis (PCA) to reduce the dimensionality of the feature vectors. The reduced feature vectors are then applied to analyze the similarity of protein sequences.

## Supplementary Information

**Additional file 1.** Supplemental Material.

## Data Availability

The source code for the latest version of FEGS package is available at https://sourceforge.net/projects/transcriptomeassembly/files/Feature%20Extraction/.
