# Supplementary material for: FEGS: a novel feature extraction model for protein sequences and its applications
Source: BMC Bioinformatics. 2021 Jun 3;22:297. doi: 10.1186/s12859-021-04223-3 (PMC8172329; doi:10.1186/s12859-021-04223-3)
Supplement: Supplementary file 1 — Additional file 1. Supplemental Material. [file 12859_2021_4223_MOESM1_ESM.pdf]

Supplemental Material

FEGS: A novel feature extraction model for protein sequences and its applications

Zengchao Mu[1, 2,†], Ting Yu [1,†], Xiaoping Liu[3,†], Hongyu Zheng, Leyi Wei[5*], Juntao Liu[2,*]
[1]Research Center for Mathematics and Interdisciplinary Sciences, Shandong University, Qingdao, 266237, China
[2]School of Mathematics and Statistics, Shandong University (Weihai), Weihai, 264209, China
[3]Hangzhou Institute for Advanced Study, University of Chinese academy of sciences
[4]Department of Radiation Oncology, Qilu Hospital, Cheeloo College of Medicine, Shandong University, Jinan, 250012, China
[5]School of Software, Shandong University, Jinan, China

1. Supplemental Notes

1.1 Selected 158 physicochemical properties

1) Signal sequence helical potential
2) Membrane-buried preference parameters
3) Average flexibility indices
4) Information value for accessibility; average fraction 23%
5) Retention coefficient in TFA
6) alpha-NH chemical shifts
7) alpha-CH chemical shifts
8) Normalized frequency of extended structure
9) A parameter defined from the residuals obtained from the best correlation of the Chou-Fasman parameter of beta-sheet
10) Average volume of buried residue
11) Frequency of the 2nd residue in turn
12) Frequency of the 4th residue in turn
13) Normalized frequency of the 2nd and 3rd residues in turn
14) Normalized hydrophobicity scales for alpha-proteins
15) Membrane preference for cytochrome b: MPH89
16) Average membrane preference: AMP07
17) Consensus normalized hydrophobicity scale
18) Hydrophobic parameter pi
19) Aperiodic indices

20) Aperiodic indices for beta-proteins
21) Aperiodic indices for alpha/beta-proteins
22) Partition energy
23) Heat capacity
24) Absolute entropy
25) Entropy of formation
26) Normalized relative frequency of alpha-helix
27) Average accessible surface area
28) Sequence frequency
29) Average relative probability of helix
30) Average relative probability of inner helix
31) Flexibility parameter for one rigid neighbor
32) Side chain interaction parameter
33) Side chain interaction parameter
34) Distance between C-alpha and centroid of side chain
35) Radius of gyration of side chain
36) Normalized frequency of beta-sheet
37) Normalized frequency of reverse turn
38) Average surrounding hydrophobicity
39) Normalized frequency of extended structure
40) Refractivity
41) Retention coefficient in HPLC
42) Retention coefficient in NaClO4
43) Effective partition energy
44) AA composition of total proteins
45) Normalized composition from animal
46) AA composition of mt-proteins from fungi and plant
47) AA composition of membrane proteins
48) Transmembrane regions of non-mt-proteins
49) Transmembrane regions of mt-proteins
50) AA composition of CYT of single-spanning proteins
51) AA composition of CYT2 of single-spanning proteins
52) AA composition of EXT of single-spanning proteins
53) AA composition of EXT2 of single-spanning proteins
54) AA composition of MEM of single-spanning proteins
55) AA composition of CYT of multi-spanning proteins
56) AA composition of EXT of multi-spanning proteins

57) 8 A contact number
58) 14 A contact number
59) Average non-bonded energy per atom
60) Long range non-bonded energy per atom
61) Average non-bonded energy per residue
62) Short and medium range non-bonded energy per residue
63) Optimized beta-structure-coil equilibrium constant
64) Optimized propensity to form reverse turn
65) Optimized transfer energy parameter
66) Optimized average non-bonded energy per atom
67) Optimized side chain interaction parameter
68) Normalized frequency of alpha-helix from CF
69) Normalized frequency of alpha-helix in alpha+beta class
70) Average gain in surrounding hydrophobicity
71) Surrounding hydrophobicity in alpha-helix
72) Average number of surrounding residues
73) Hydrophobicity
74) Relative frequency in reverse-turn
75) Weights for alpha-helix at the window position of -3
76) Weights for alpha-helix at the window position of 0
77) Weights for alpha-helix at the window position of 3
78) Weights for beta-sheet at the window position of 0
79) Weights for coil at the window position of -1
80) Weights for coil at the window position of 0
81) Weights for coil at the window position of 1
82) Weights for coil at the window position of 5
83) Average reduced distance for side chain
84) Average relative fractional occurrence in AR (i)
85) Average relative fractional occurrence in A0 (i-1)
86) Value of theta (i)
87) Transfer free energy from vap to chx
88) Accessible surface area
89) Information measure for middle helix
90) Mean area buried on transfer
91) Principal component I
92) Principal component III
93) Principal component IV

94) Normalized frequency of isolated helix
95) Normalized frequency of extended structure
96) Normalized frequency of chain reversal R
97) Normalized frequency of coil
98) Relative population of conformational state A
99) Relative population of conformational state C
100) Transfer free energy to lipophilic phase
101) Average interactions per side chain atom
102) Hydration potential
103) Principal property value z1
104) Principal property value z2
105) Principal property value z3
106) Activation Gibbs energy of unfolding
107) Normalized positional residue frequency at helix termini C3
108) Normalized flexibility parameters
109) Free energy in alpha-helical region
110) Free energy in beta-strand conformation
111) p-Values of thermophilic proteins based on the distributions of B values
112) Distribution of amino acid residues in the 18 non-redundant families of thermophilic proteins
113) Distribution of amino acid residues in the alpha-helices in thermophilic mesophilic proteins
114) Hydropathy scale based on self-information values in the two-state model (16% accessibility)
115) Hydropathy scale based on self-information values in the two-state model (25% accessibility)
116) Hydropathy scale based on self-information values in the two-state model (50% accessibility)
117) Alpha-helix propensity derived from designed sequences
118) Surface composition of amino acids in intracellular proteins of thermophiles
119) Surface composition of amino acids in intracellular proteins of mesophiles
120) Surface composition of amino acids in nuclear proteins
121) Interior composition of amino acids in intracellular proteins of thermophiles
122) Interior composition of amino acids in intracellular proteins of mesophiles
123) Interior composition of amino acids in extracellular proteins of mesophiles
124) Entire chain composition of amino acids in intracellular proteins of thermophiles
125) Entire chain composition of amino acids in extracellular proteins of mesophiles

126) Entire chain compositino of amino acids in nuclear proteins
127) Volumes including the crystallographic waters using the ProtOr
128) Volumes not including the crystallographic waters using the ProtOr
129) Hydrophobicity scales
130) Hydrophobicity coefficient in RP-HPLC
131) Hydrophobicity coefficient in RP-HPLC
132) Interactivity scale obtained from the contact matrix
133) Interactivity scale obtained by maximizing the mean of correlation coefficient over single-domain globular proteins
134) Interactivity scale obtained by maximizing the mean of correlation coefficient over pairs of sequences sharing the TIM barrel fold
135) Linker propensity index
136) Linker propensity from 2-linker dataset
137) Linker propensity from medium dataset
138) Linker propensity from long dataset
139) Linker propensity from helical (annotated by DSSP) dataset
140) Linker propensity from non-helical (annotated by DSSP) dataset
141) Linker index
142) Mean volumes of residues buried in protein interiors
143) Average volumes of residues
144) Hydrostatic pressure asymmetry index
145) Average internal preferences
146) Apparent partition energies calculated from Janin index
147) Apparent partition energies calculated from Chothia index
148) Weights from the IFH scale
149) Hydrophobicity index
150) NNEIG index
151) SWEIG index
152) PRIFT index
153) ALTFT index
154) ALTLS index
155) TOTFT index
156) TOTLS index
157) Relative partition energies derived by the Bethe approximation
158) Hydrophobicity index

1.2 Accession numbers of the 50 beta-globin proteins

Human (AAA16334.1), Pigeon (P11342.1), Goshawk (P08851.1), Black bear (P68012.1)), Lesser panda (P18982.1), Asiatic elephant (P02084.1), Giant panda (P18983.2), African elephant (P02085.1), Sheep (P02075.2), Tortoise (P83123.3), Duck (P02114.2), Grivet (P02028.1), Mallard (P02115.1), Gorilla (P02024.2), Goose (P02117.1), Shark (P02143.1), Rat (CAA33114.1), Hippopotamus (P19016.1), Penguin (P80216.1), Horse (P02062.1), Swift (P15165.1), Gibbon (P02025.1), Coyote (P60525.1), Whale (P18984.1), Catfish (O13163.2), Bat (P24660.1), Bison (P09422.1), Red fox (P21201.1), Swan (P68945.1), Marmot (P08853.1), Buffalo (P67820.1), Salmon (Q91473.3), Dog (P60524.1), Sparrow (P07406.1), Chimpanzee (P68873.2), Pheasant (P02113.1), Dolphin (P18990.1), Flamingo (P02121.1), Goldfish (P02140.1), Pig (P02067.3), Polar bear (P68011.1), Dragonfish (ADD73488.1), Rhinoceros (P09907.1), Parakeet (P21668.1), Chicken (P02112.2), Zebra (P67824.1), Wolf (P60526.1), Cod (O13077.2), Turtle (P13274.1) and Langur (P02032.1).

1.3 Clusters of protein sequences based on taxonomy

1.3.1 Clusters of protein sequences in data set 1. The 50 beta-globin protein sequences belong to four groups, Aves, Reptilia, Pisces, and Mammals as follows.
Aves: Goshawk, Mallard, Duck, Goose, Penguin, Swift, Swan, Chicken, Pigeon, Sparrow, Pheasant, Flamingo, Parakeet;
Reptilia: Turtle, Tortoise;
Pisces: Catfish, Goldfish, Shark, Salmon, Dragonfish, Cod;
Mammals: Human, Lesser panda, Giant panda, Sheep, Rat, Coyote, Bison, Dog, Buffalo, Chimpanzee, Dolphin, Polar bear, Rhinoceros, Wolf, Black bear, Asiatic elephant, African elephant, Grivet, Gorilla, Hippopotamus, Gibbon, Horse, Whale, Bat, Langur, Zebra, Red fox, Marmot, Pig.

1.3.2 Clusters of protein sequences in data set 2. The 27 antifreeze protein sequences belong to six groups, Choristoneura fumiferana (CF), Tenebrio molitor (TM), Hypogastrura harveyi (HH), Dorcus curvidens binodulosus (DCB), Microdera dzhungarica punctipennis (MDP) and Dendroides canadensis (DC) as follows.
CF: CF11181767, CF11181763, CF12382208;
TM: TM82494434, TM82494426, TM82494418, TM2411490, TM60593179, TM5882241, TM5882233, TM78173115, TM78173113, TM78173111, TM78173109, TM77812622, TM77812618, TM77812620, TM2411494, TM21465961;
HH: HH77808090;
DCB: DCB1308442, DCB1308440, DCB1308436;
MDP: MDP8220717, MDP8220719;
DC: DC9280348, DC9280340;

1.3.3 Clusters of protein sequences in data set 3. The 40 coronavirus spike protein sequences belong to three groups, Alphacoronavirus, Betacoronavirus and Gammacoronavirus as follows.

Alphacoronavirus: FIPV-1146, FCoV-1683, PEDVC, TGEVT, TGEVF, CECoV;

Betacoronavirus: MHVM, MHVB, MHVA, MHVD, RtCoV, BCoVF, BCoVM, BCoVL, BCoVT, HCoV-OC43, Tor2, BJ01, NS-1, GD01, Frankfurt 1, Urbani, TC1, CDC, GZ02, QXC1, Sino1-11, TJF, NIMH-1598, HN023, NY-PV08438, NJ-CDC-3592, CA-CZB-1104;

Gammacoronavirus: IBV, IBV-6/82, IBVD, IBVC, IBVA, IBVB, IBVH.

1.3.4 Clusters of protein sequences in data set 4. The 25 protein sequences belong to six groups, Mammalian TF, Mammalian LF, Amphibian, Pleuronectiformes, Beloniformes, Salmoniformes as follows.

Mammalian TF: Human TF, Rabit TF, Rat TF, Cow TF;

Mammalian LF: Buffalo LF, Cow LF, Goat LF, Camel LF, Pig LF, Human LF, Mouse LF, Possum TF;

Amphibian: Frog TF;

Pleuronectiformes: Japanese flounder TF;

Beloniformes: Medaka TF;

Salmoniformes: Atlantic salmon TF, Brown trout TF, Lake trout TF, Brook trout TF, Japanese char TF, Chinook salmon TF, Coho salmon TF, Sockeye salmon TF, Rainbow trout TF, Amago salmon TF；

1.3.5 Clusters of protein sequences in data set 5. The 114 protein sequences belong to four groups, HRV-A, HRV-B, HRV-C, and HEV-C as follows.

HRV-A: AY751783 A hrv 39, DQ473491 A hrv 41, DQ473492 A hrv 73, DQ473493 A hrv 15, DQ473494 A hrv 74, DQ473496 A hrv 49, DQ473497 A hrv 23, DQ473499 A hrv 44, DQ473500 A hrv 59, DQ473504 A hrv 88, DQ473505 A hrv 36, DQ473506 A hrv 46, DQ473507 A hrv 53, DQ473508 A hrv 28, DQ473510 A hrv 75, DQ473511 A hrv 55, EF173414 A hrv 11, EF173415 A hrv 12, FJ445111 A hrv 01, FJ445113 A hrv 08, FJ445114 A hrv 09 f01, FJ445115 A hrv 09 f02, FJ445116 A hrv 13, FJ445117 A hrv 13 f03, FJ445118 A hrv 18, FJ445119 A hrv 19, FJ445121 A hrv 21, FJ445122 A hrv 22, FJ445123 A hrv 25, FJ445125 A hrv 29, FJ445126 A hrv 31, FJ445127 A hrv 32, FJ445128 A hrv 33, FJ445129 A hrv 40, FJ445131 A hrv 43, FJ445132 A hrv 45, FJ445133 A hrv 47, FJ445134 A hrv 49 f04, FJ445135 A hrv 50, FJ445136 A hrv 51, FJ445138 A hrv 54, FJ445139 A hrv 54 f05, FJ445140 A hrv 56, FJ445141 A hrv 57, FJ445142 A hrv 58, FJ445143 A hrv 60, FJ445144 A hrv 61, FJ445145 A hrv 62, FJ445146 A hrv 63, FJ445147 A hrv 65, FJ445148 A hrv 66, FJ445149 A hrv 67, FJ445152 A hrv 71, FJ445154 A hrv 77, FJ445156 A hrv 80, FJ445157 A hrv 81, FJ445158 A hrv 81 f06, FJ445160 A hrv 82, FJ445163 A hrv 85, FJ445165 A hrv 89 f09, FJ445166 A hrv 89 f08, FJ445167 A hrv 90, FJ445170 A hrv 95, FJ445171 A hrv 96, FJ445173 A hrv 98, FJ445175 A hrv 100, FJ445176 A hrv 07, FJ445177 A hrv 09, FJ445178 A hrv 10, FJ445179 A hrv 30, FJ445180 A hrv 38, FJ445181 A hrv 64, FJ445182 A hrv 76, FJ445183 A hrv 78, FJ445184 A hrv 89, FJ445185 A hrv 94, FJ445189 A hrv 34, FJ445190 A hrv 24, L24917 A hrv 16, X02316 A hrv 02;

HRV-B: DQ473485 B hrv 03, DQ473486 B hrv 06, DQ473488 B hrv 48, DQ473489 B hrv

70, DQ473490 B hrv 04, EF173420 B hrv 17, EF173423 B hrv 37, EF173425 B hrv 93, FJ445112 B hrv 05, FJ445124 B hrv 26, FJ445137 B hrv 52 f10, FJ445151 B hrv 69, FJ445153 B hrv 72, FJ445155 B hrv 79, FJ445161 B hrv 83, FJ445162 B hrv 84, FJ445164 B hrv 86, FJ445168 B hrv 91, FJ445169 B hrv 92, FJ445172 B hrv 97, FJ445174 B hrv 99, FJ445186 B hrv 27, FJ445187 B hrv 35, FJ445188 B hrv 52, L05355 B hrv 14;
HRV-C: EF077279 C nat001, EF077280 C nat045, EF186077 C qpm, EF582385 C c024, EF582386 C c025, EF582387 C c026;
HEV-C: AF499637 HEV cva 13, AF546702 HEV cva 21, V01149 HEV pv 1m.

2. Supplemental Tables

Table S1. The information for 27 antifreeze proteins

Sequence name	Accession	Species
DC9280348	AAF86363.1	Dendroides Canadensis
DC9280340	AAF86359.1	Dendroides canadensis
TM82494434	ABB79834.1	Tenebrio molitor
TM82494426	ABB79830.1	Tenebrio molitor
TM82494418	ABB79826.1	Tenebrio molitor
TM2411490	AAB70750.1	Tenebrio molitor
TM60593179	AAX28872.1	Tenebrio molitor
TM5882241	AAD55260.1	Tenebrio molitor
TM5882233	AAD55256.1	Tenebrio molitor
TM78173115	ABB29474.1	Tenebrio molitor
TM78173113	ABB29473.1	Tenebrio molitor
TM78173111	ABB29472.1	Tenebrio molitor
TM78173109	ABB29471.1	Tenebrio molitor
TM77812622	ABB03885.1	Tenebrio molitor
TM77812618	ABB03883.1	Tenebrio molitor
TM77812620	ABB03884.1	Tenebrio molitor
TM2411494	AAB70752.1	Tenebrio molitor
TM21465961	1L1IA	Tenebrio Molitor
MDP8220717	AAW67979.1	Microdera dzhungarica punctipennis
MDP8220719	AAW67980.1	Microdera dzhungarica punctipennis
DCB1308442	BAF43605.1	Dorcus curvidens binodulosus
DCB1308440	BAF43604.1	Dorcus curvidens binodulosus
DCB1308436	BAF43602.1	Dorcus curvidens binodulosus
CF11181767	AAF86612.1	Choristoneura fumiferana
CF11181763	AAF86610.1	Choristoneura fumiferana
CF12382208	ABI17375.1	Choristoneura fumiferana
HH77808090	ABB03725.1	Hypogastrura harveyi

Table S2. The information of 40 coronavirus spike proteins.

No.	Accession	Abbreviation	Name
1	P10033	FIPV-1146	Feline infectious peritonitis virus strain 79-1146
2	Q66928	FCoV-1683	Feline coronavirus strain 79-1683
3	Q91AV1	PEDVC	Porcine epidemic diarrhea virus strain CV777
4	Q9DY22	TGEVT	Transmissible gastroenteritis virus strain TO14
5	P18450	TGEVF	Porcine transmissible gastroenteritis coronavirus strain FS772/70
6	P36300	CECoV	Canine enteric coronavirus strain INSAVC-1
7	Q82666	IBV	Infectious bronchitis virus
8	P05135	IBV-6/82	Avian infectious bronchitis virus strain 6/82
9	P12722	IBVD	Avian infectious bronchitis virus strain D274
10	Q64930	IBVC	Infectious bronchitis virus strain CU-T2
11	Q82624	IBVA	Infectious bronchitis virus strain Ark99
12	P11223	IBVB	Avian infectious bronchitis virus strain Beaudette
13	Q98Y27	IBVH	Infectious bronchitis virus strain H52
14	Q9J3E7	MHVM	Murine hepatitis virus strain ML-10
15	Q83331	MHVB	Murine hepatitis virus strain Berkeley
16	P11224	MHVA	Murine hepatitis virus strain A59
17	O55253	MHVD	Murine hepatitis virus strain DVIM
18	Q9IKD1	RtCoV	Rat coronavirus strain 681
19	P25190	BCoVF	Bovine coronavirus strain F15
20	P15777	BCoVM	Bovine coronavirus strain Mebus
21	Q9QAR5	BCoVL	Bovine coronavirus strain LSU-94LSS-051
22	Q91A26	BCoVT	Bovine enteric coronavirus 98TXSF-110-ENT
23	P36334	HCoV-OC43	Human coronavirus strain OC43
24	AAP41037	Tor2	SARS coronavirus Tor2
25	AAP30030	BJ01	SARS coronavirus BJ01
26	AAR91586	NS-1	SARS coronavirus NS-1
27	AAP51227	GD01	SARS coronavirus GD01
28	AAP33697	Frankfurt 1	SARS coronavirus Frankfurt 1
29	AAP13441	Urbani	SARS coronavirus Urbani
30	AAQ01597	TC1	SARS coronavirus Taiwan TC1
31	AAU81608	CDC	SARS Coronavirus CDC#200301157
32	AAS00003	GZ02	SARS coronavirus GZ02
33	AAR86788	QXC1	SARS coronavirus ShanghaiQXC1
34	AAR23250	Sino1-11	SARS coronavirus Sino1-11
35	AAT76147	TJF	SARS coronavirus TJF
36	QJQ27878	NIMH-1598	2019 novel coronavirus NIMH-1598
37	QJG65952	HN023	2019 novel coronavirus HN023
38	QJD23474	NY-PV08438	2019 novel coronavirus NY-PV08438
39	QIZ15969	NJ-CDC-3592	2019 novel coronavirus NJ-CDC-3592
40	QJS54742	CA-CZB-110	2019 novel coronavirus CA-CZB-1104

Table S3. The information for transferrin sequences from 25 vertebrates

Name	Species	Accession	Length
Human TF	Homo sapiens	S95936	698
Rabbit TF	Oryctolagus coniculus	X58533	695
Rat TF	Rattus norvegicus	D38380	698
Cow TF	Bos Taurus	U02564	704
Buffalo LF	Bubahts arnee	AJ005203	708
Cow LF	Bos Taurus	X57084	708
Goat LF	Copra hircus	X78902	708
Camel LF	Camelus dromedarius	AJ131674	708
Pig LF	Sus scrofa	M92089	704
Human LF	Homo sapiens	NM_002343	710
Mouse LF	Mus musculus	NM_008522	707
Possum TF	Trichosurus vulpecula	AF092510	711
Frog TF	Xenopus laevis	X54530	702
Medaka TF	Oryzias latipes	D64033	690
Japanese flounder TF	Paralichthys olivaceus	D88801	685
Atlantic salmon TF	Salmo salar	L20313	690
Brown trout TF	Salmo trutta	D89091	691
Lake trout TF	Salvelinus namaycush	D89090	691
Brook trout TF	Salvelinus fontinalis	D89089	691
Japanese char TF	Salvelinus pluvius	D89088	691
Chinook salmon TF	Oncorhynchus tshawytscha	AH008271	677
Coho salmon TF	Oncorhynchus hisutch	D89084	691
Sockeye salmon TF	Oncorhynchus nerka	D89085	691
Rainbow trout TF	Oncorhynchus mykiss	D89083	691
Amago salmon TF	Oncorhynchus masou	D89086	691

3. Supplemental Figures

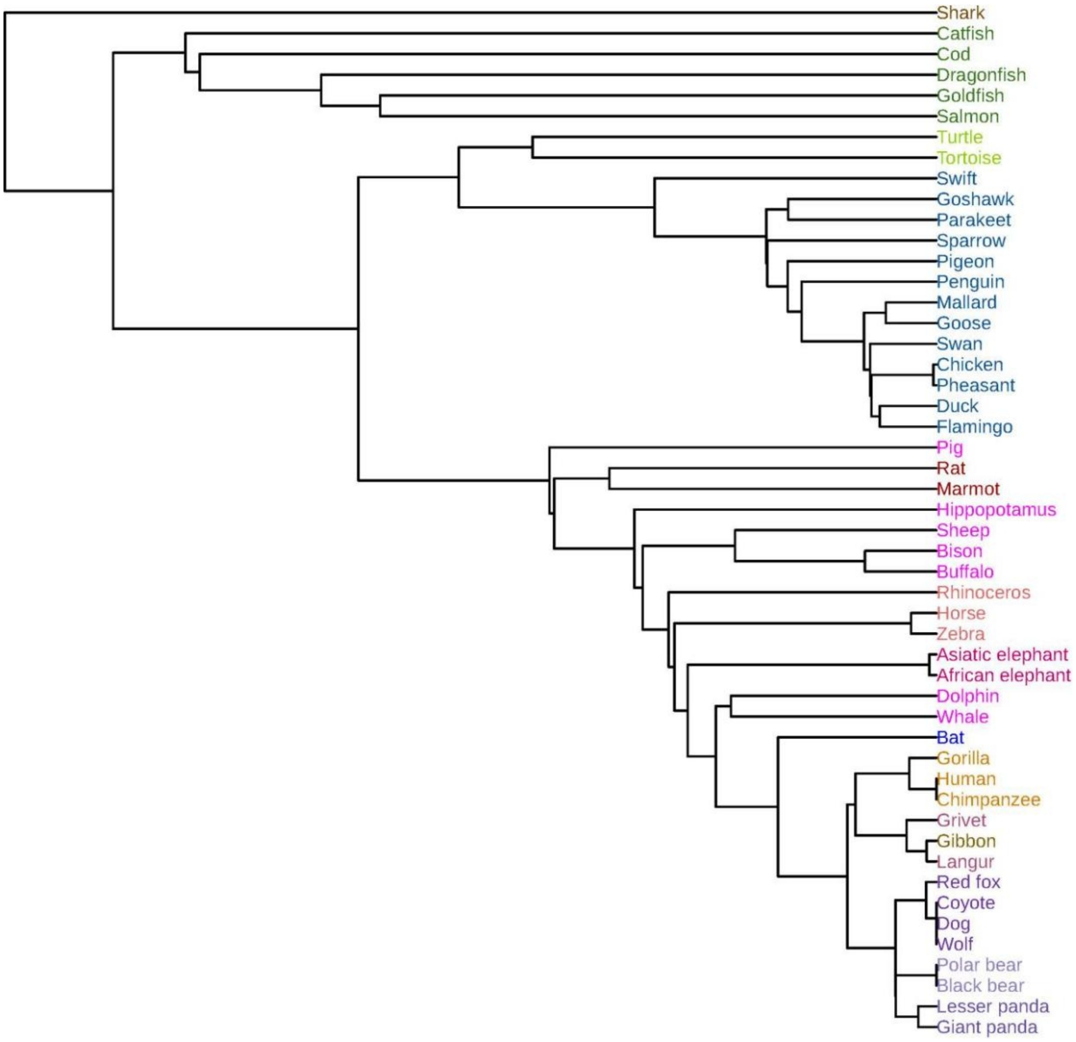

Fig. S1. The phylogenetic tree of 50 beta-globin protein sequences constructed by *k*-mer natural vector.

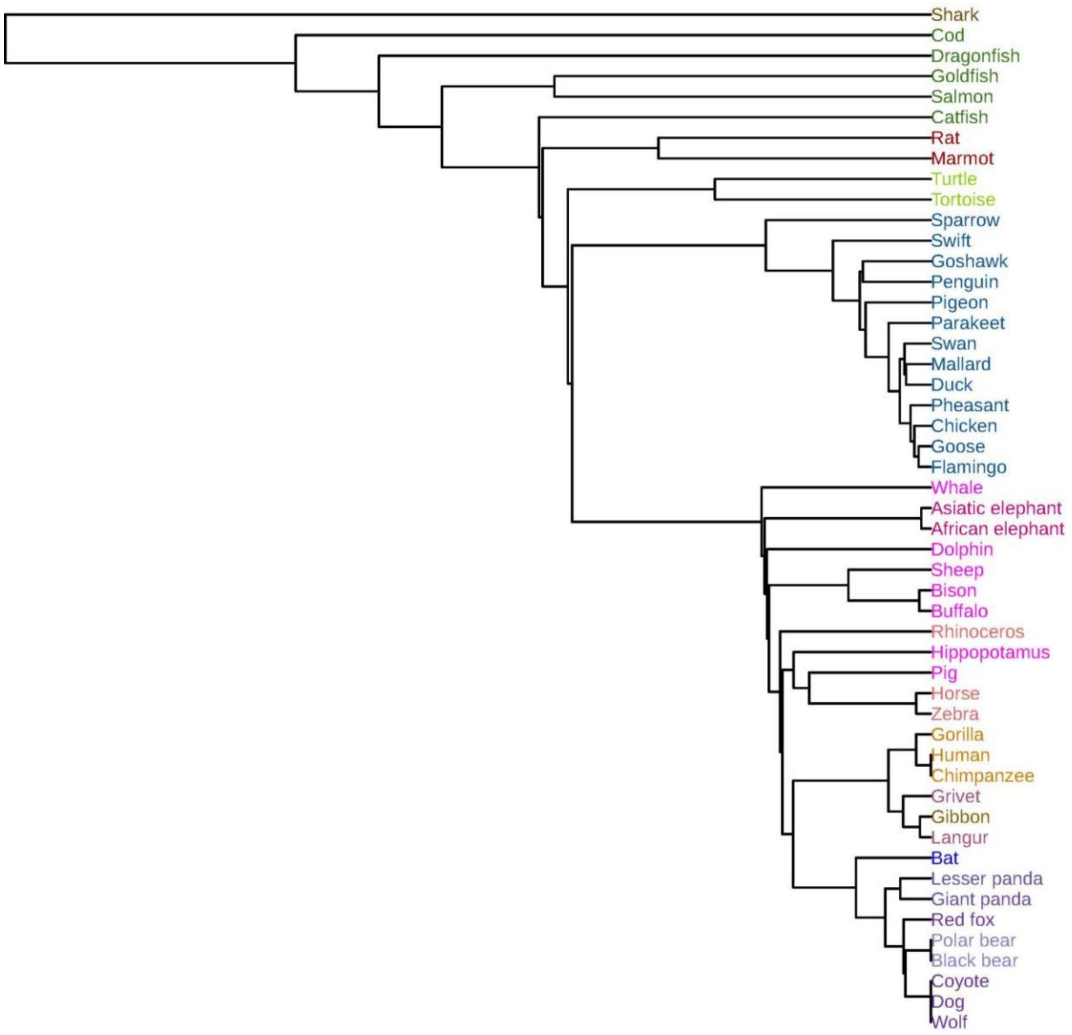

Fig. S2. The phylogenetic tree of 50 beta-globin protein sequences constructed by PseAAC .

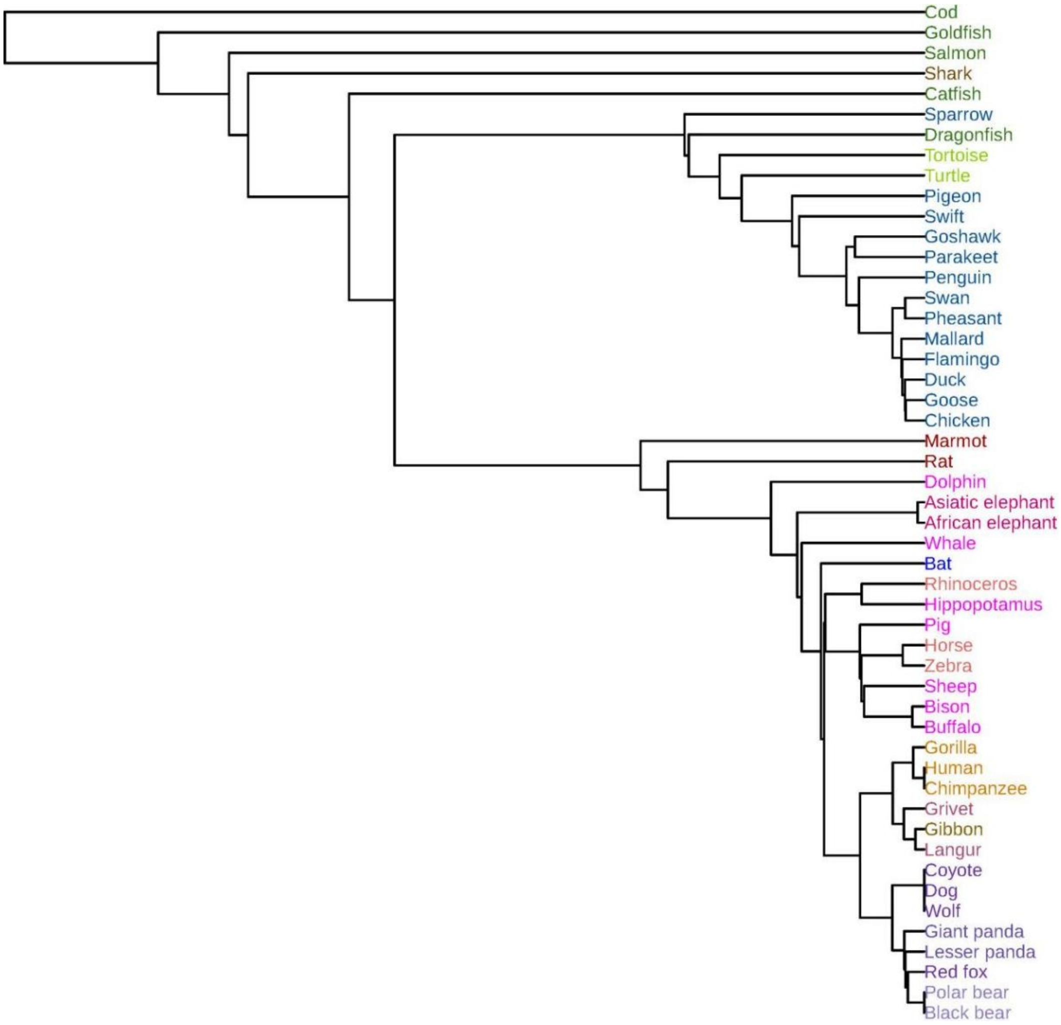

Fig. S3. The phylogenetic tree of 50 beta-globin protein sequences constructed by averaged property factor.

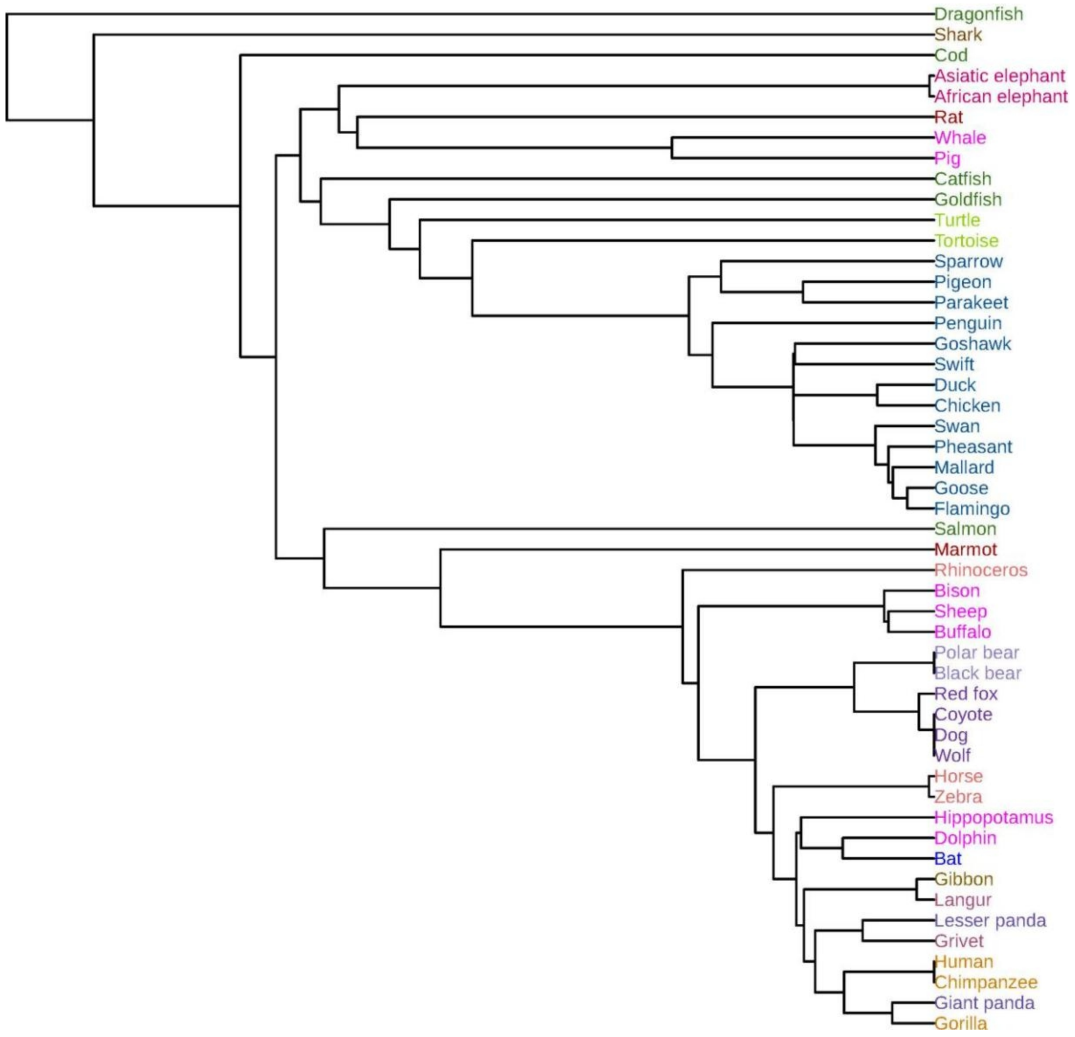

Fig. S4. The phylogenetic tree of 50 beta-globin protein sequences constructed by natural vector.

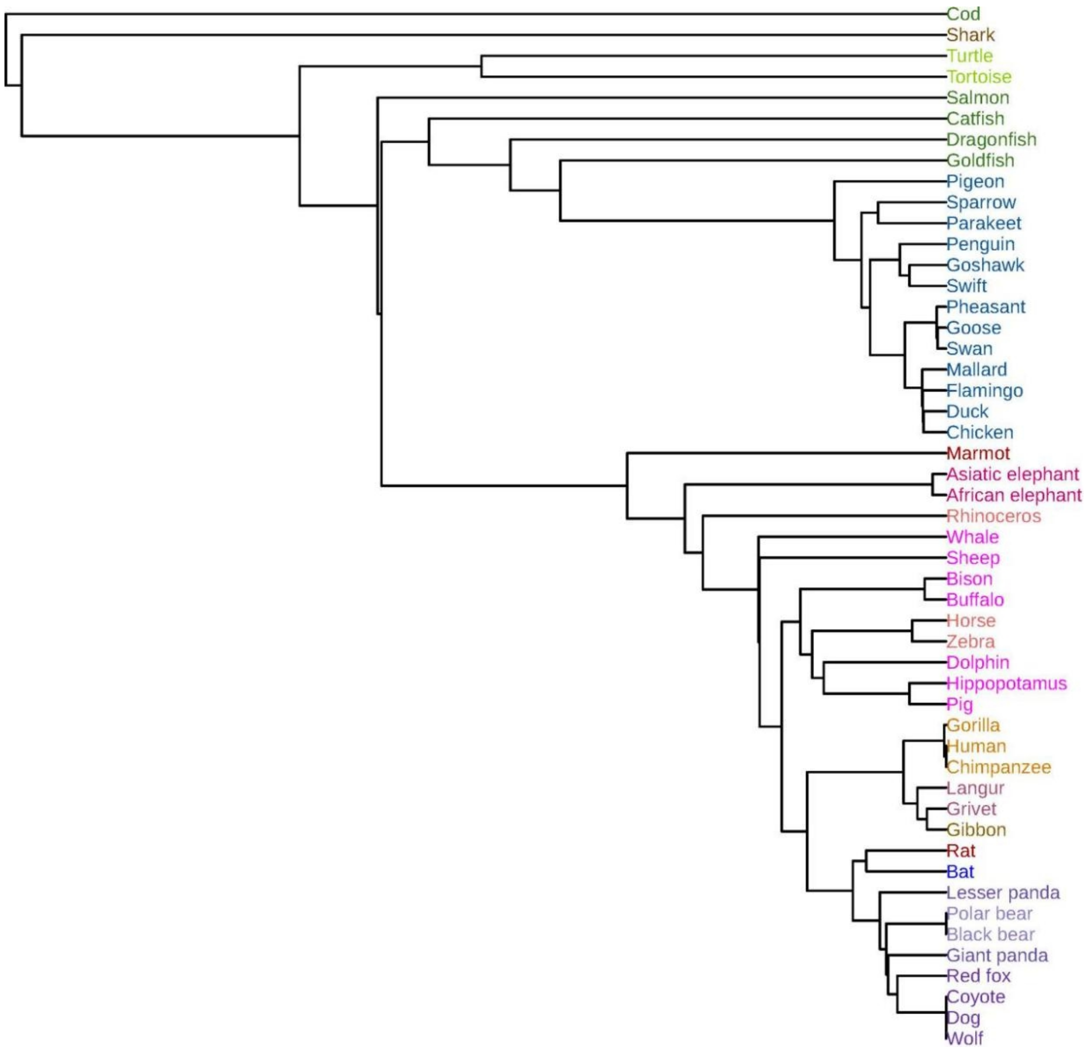

Fig. S5. The phylogenetic tree of 50 beta-globin protein sequences constructed by protein map.

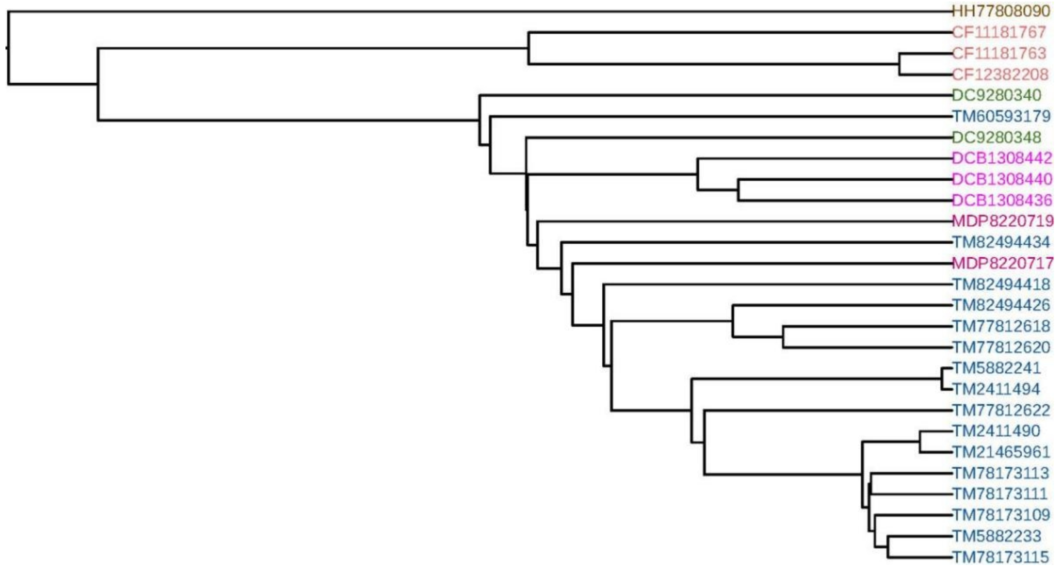

Fig. S6. The phylogenetic tree of the 27 AFPs constructed by *k*-mer natural vector.

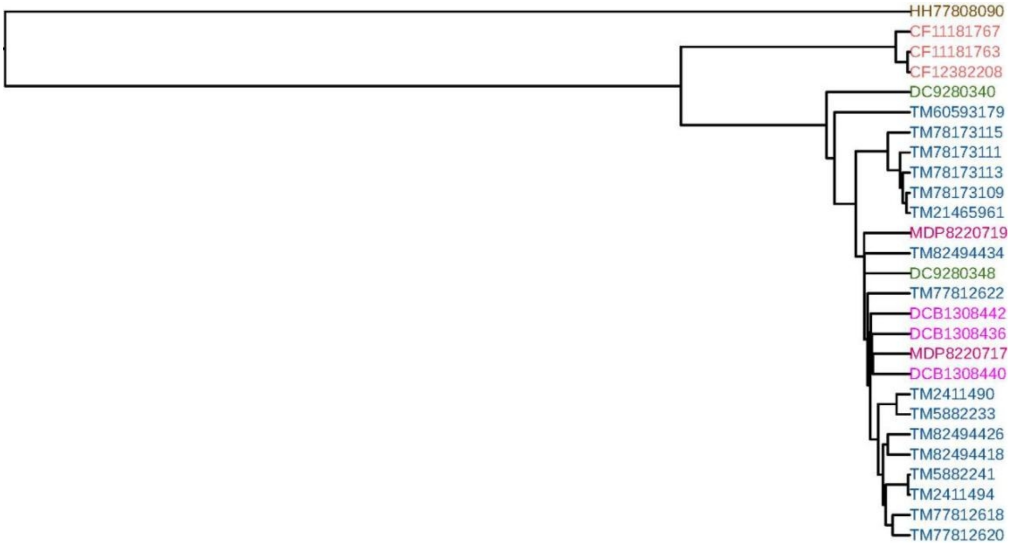

Fig. S7. The phylogenetic tree of the 27 AFPs constructed by PseAAC.

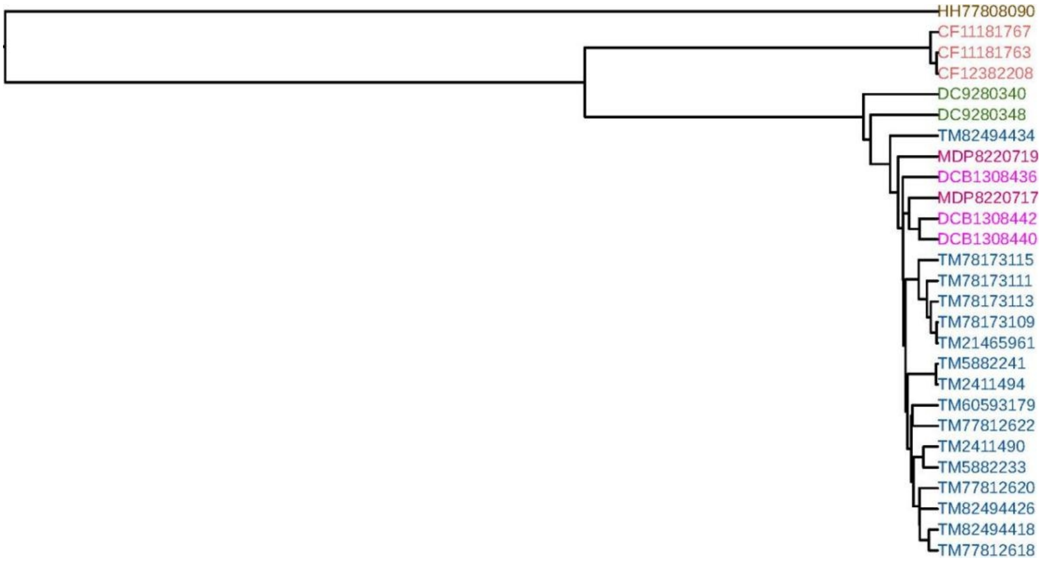

Fig. S8. The phylogenetic tree of the 27 AFPs constructed by averaged property factor.

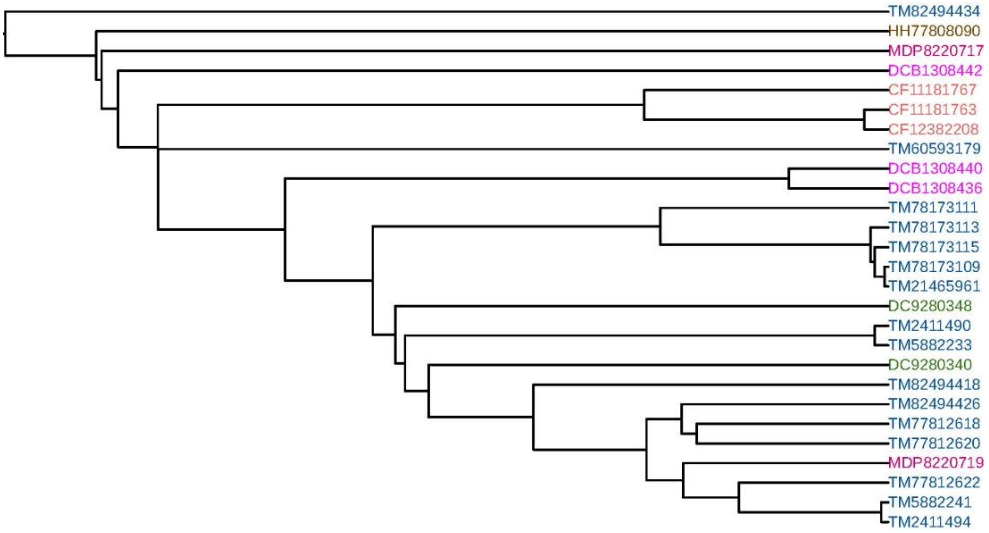

Fig. S9. The phylogenetic tree of the 27 AFPs constructed by natural vector.

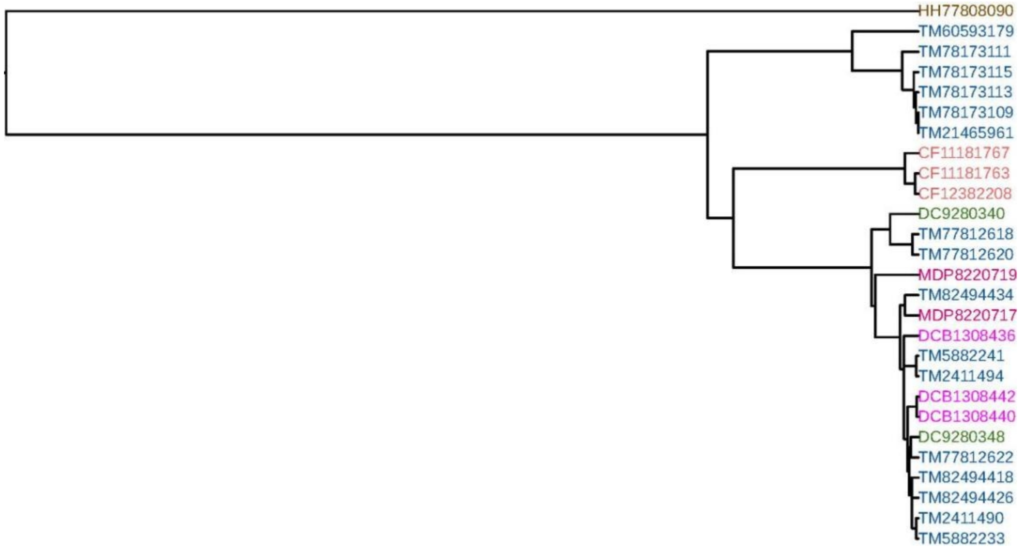

Fig. S10. The phylogenetic tree of the 27 AFPs constructed by protein map.

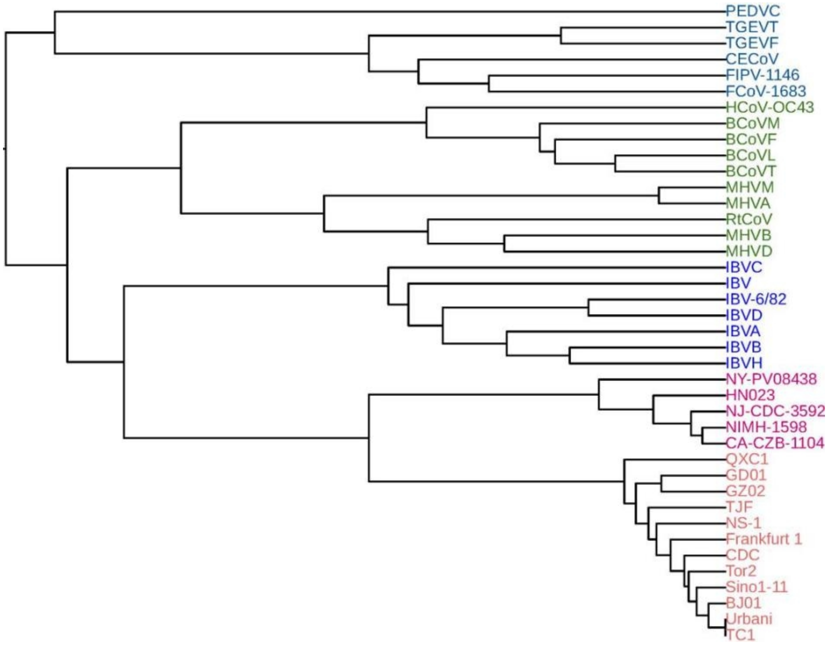

Fig. S11. The phylogenetic tree of the 40 coronavirus spike proteins constructed by *k*-mer natural vector.

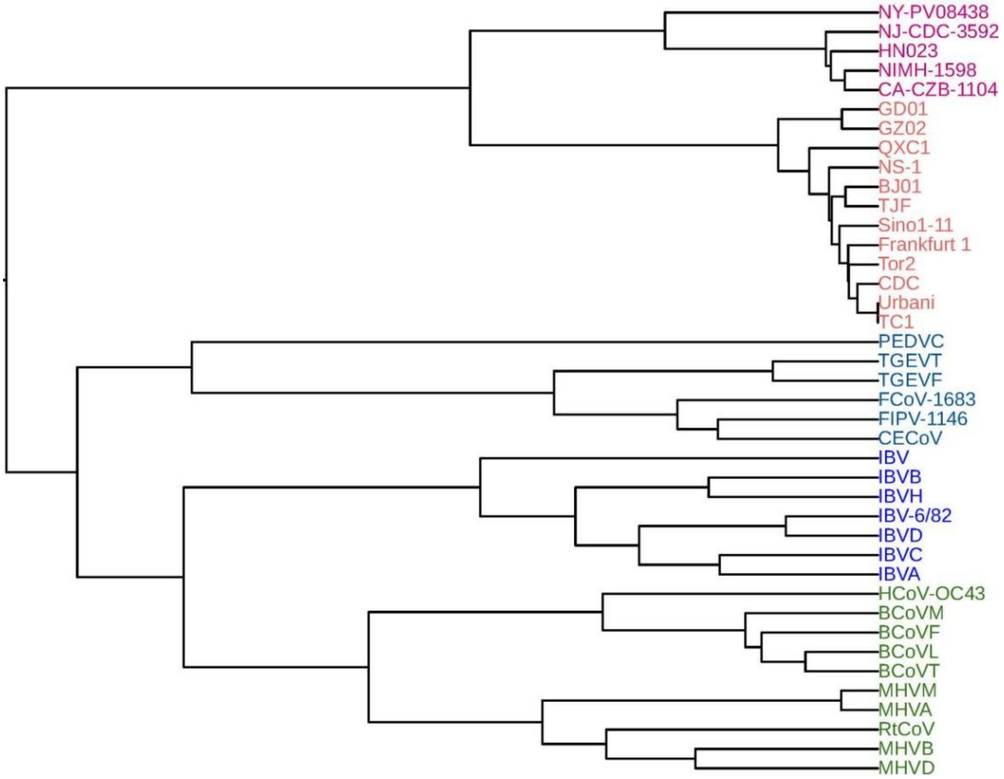

Fig. S12. The phylogenetic tree of the 40 coronavirus spike proteins constructed by PseAAC .

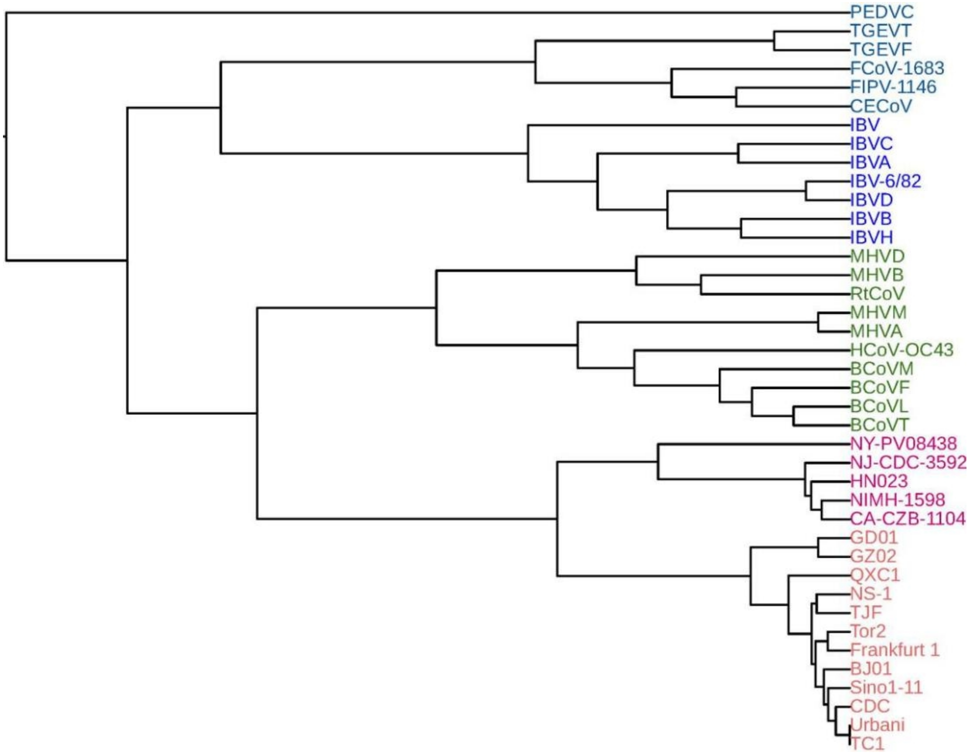

Fig. S13. The phylogenetic tree of the 40 coronavirus spike proteins constructed by averaged property factor.

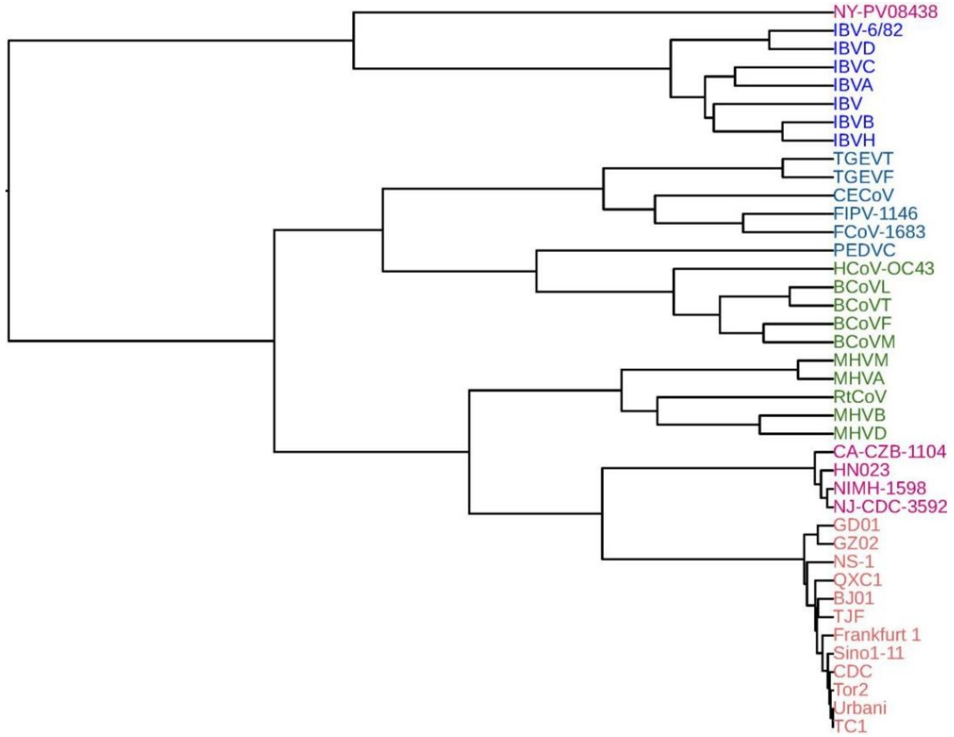

Fig. S14. The phylogenetic tree of the 40 coronavirus spike proteins constructed by natural vector.

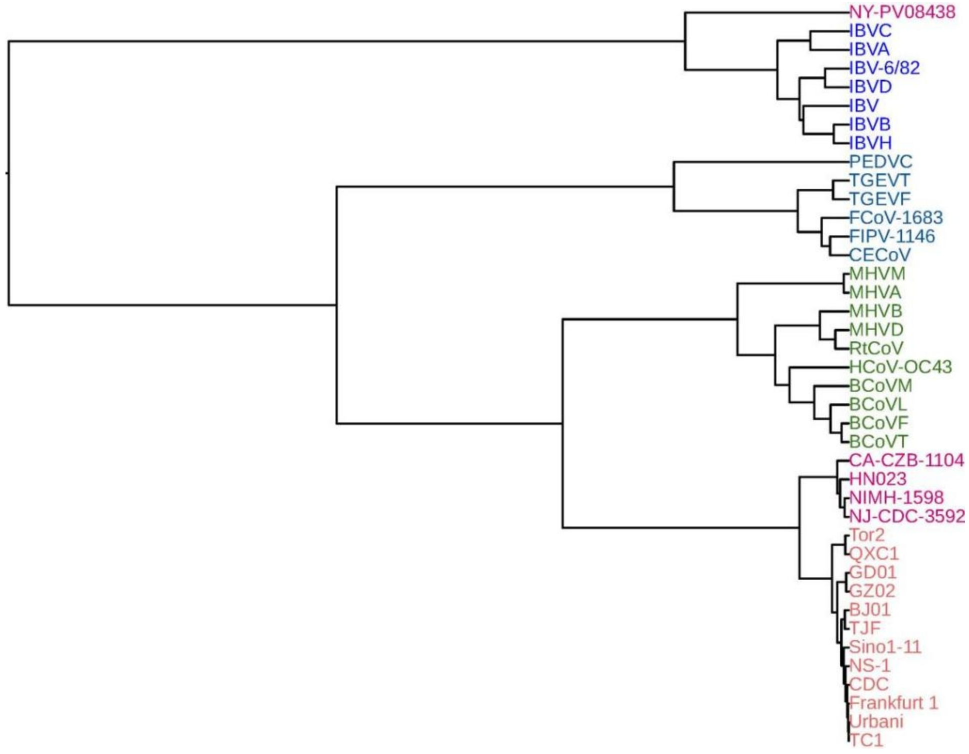

Fig. S15. The phylogenetic tree of the 40 coronavirus spike proteins constructed by protein map.

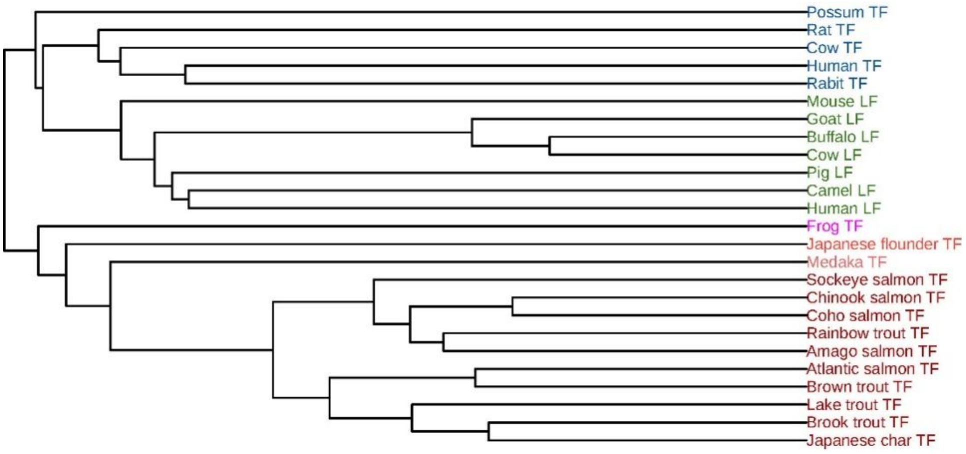

Fig. S16. The phylogenetic tree of the 25 TFs constructed by *k*-mer natural vector.

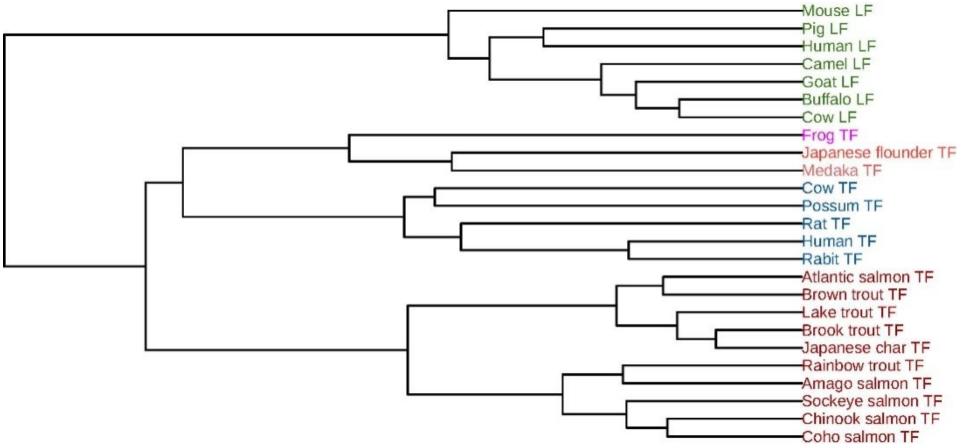

Fig. S17. The phylogenetic tree of the 25 TFs constructed by PseAAC.

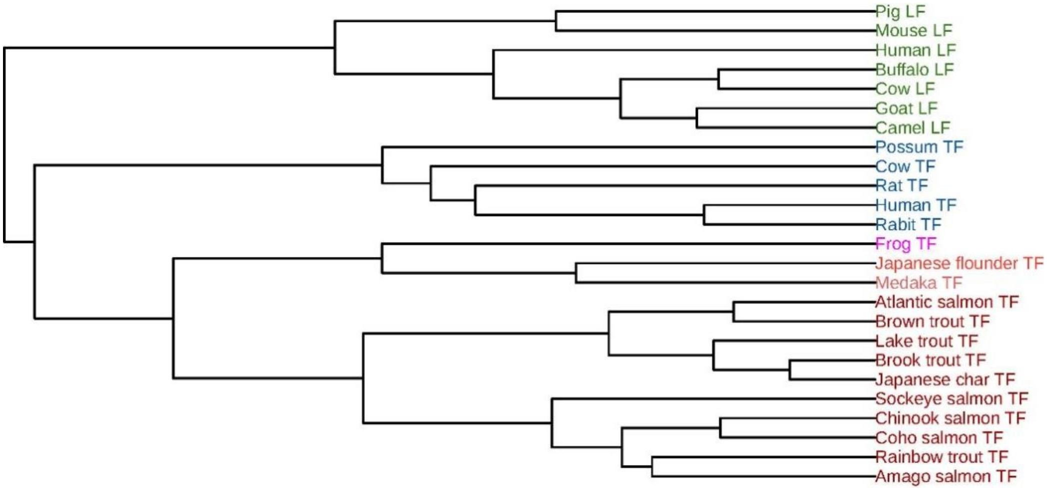

Fig. S18. The phylogenetic tree of the 25 TFs constructed by averaged property factor.

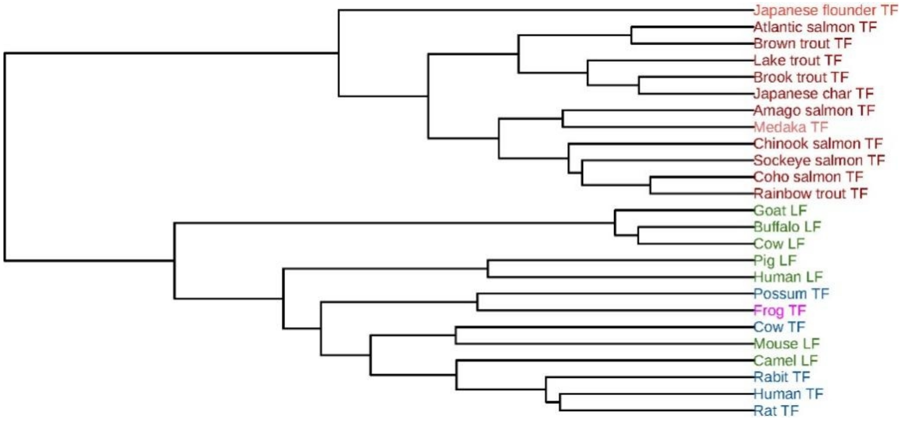

Fig. S19. The phylogenetic tree of the 25 TFs constructed by natural vector.

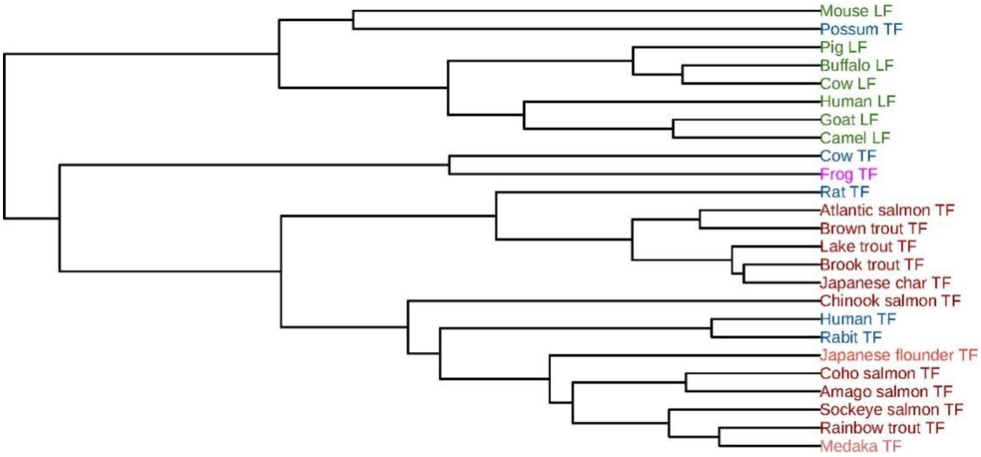

Fig. S20. The phylogenetic tree of the 25 TFs constructed by protein map.

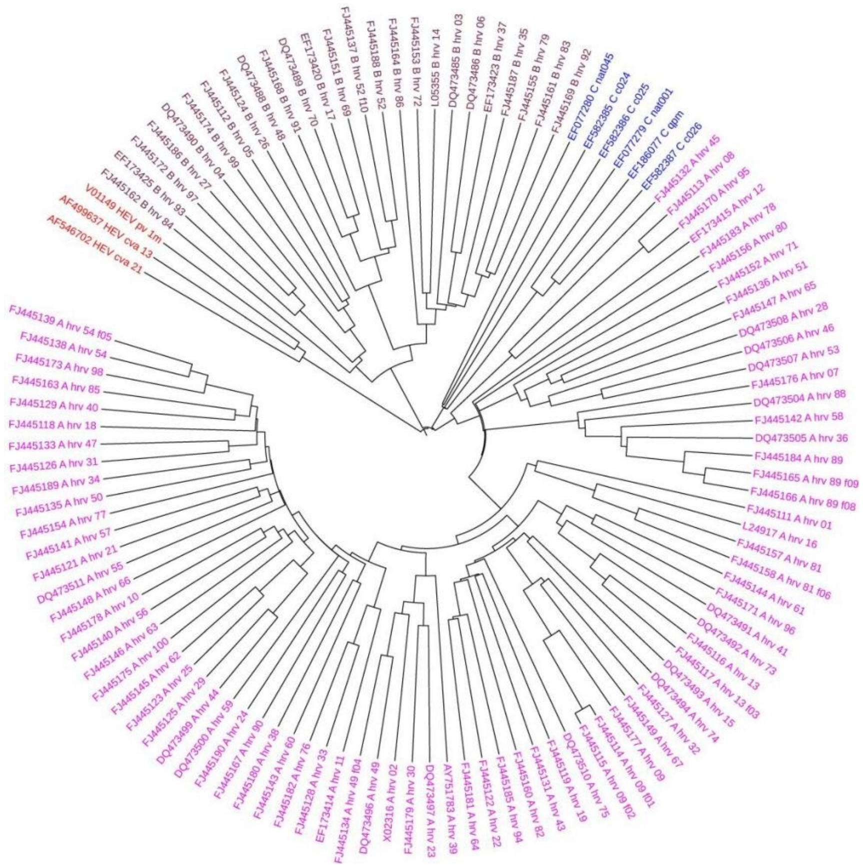

Fig. S21. The phylogenetic tree of the HRVs constructed by *k*-mer natural vector.

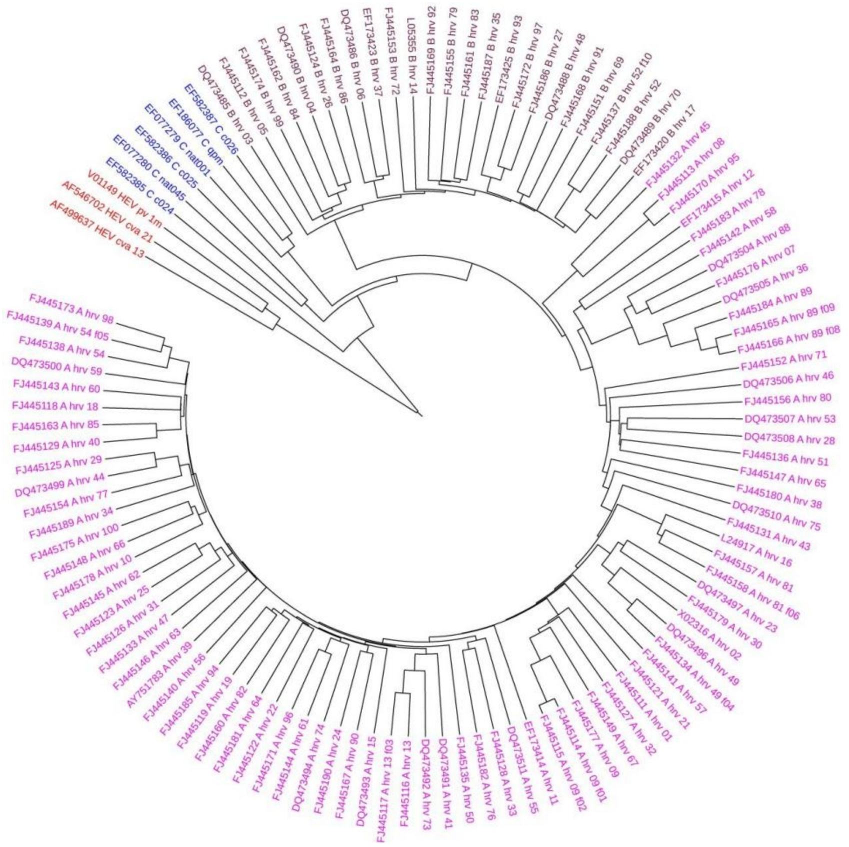

Fig. S22. The phylogenetic tree of the HRVs constructed by PseAAC.

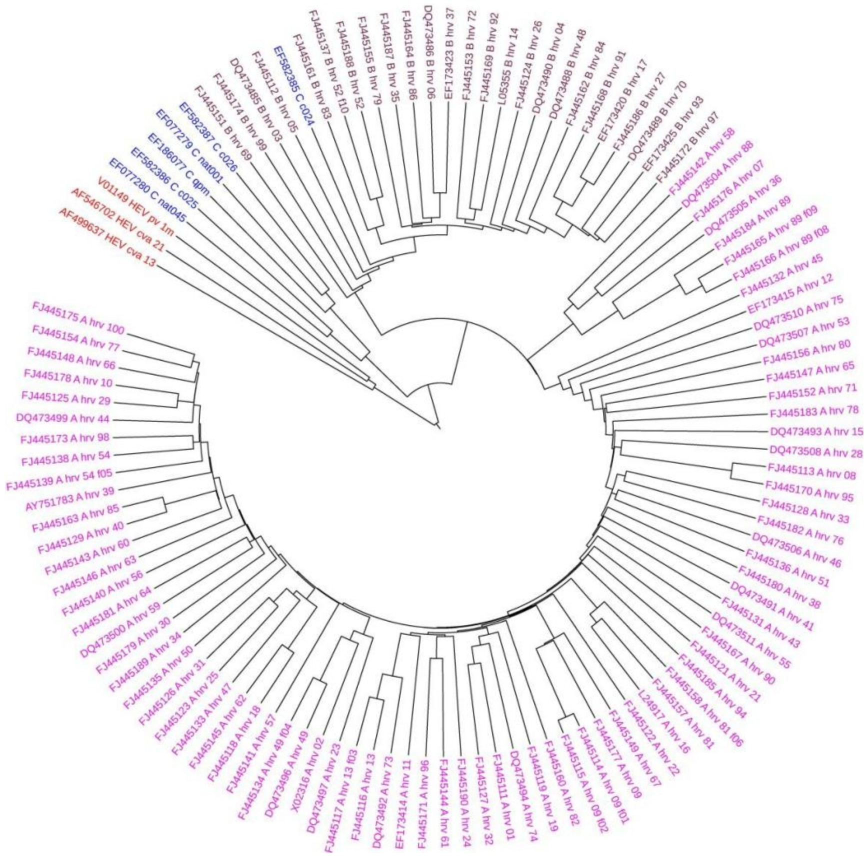

Fig. S23. The phylogenetic tree of the HRVs constructed by averaged property factor.

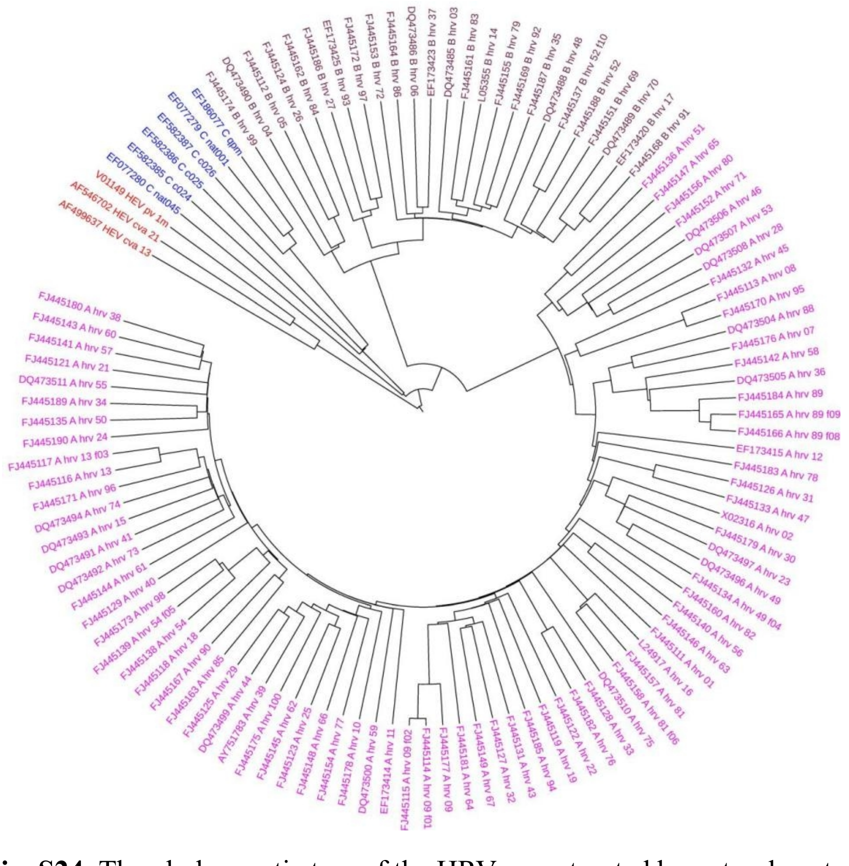

Fig. S24. The phylogenetic tree of the HRVs constructed by natural vector.

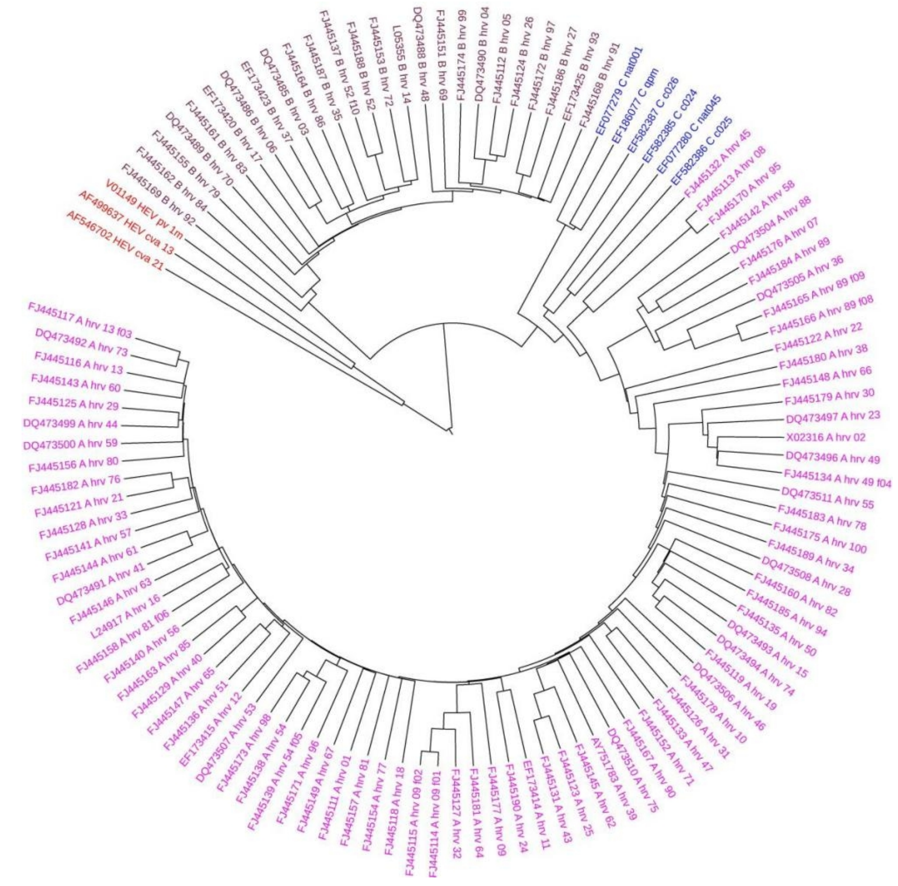

Fig. S25. The phylogenetic tree of the HRVs constructed by protein map.
